# A Petri Net Model of Granulomatous Inflammation: Implications for IL-10 Mediated Control of *Leishmania donovani* Infection

**DOI:** 10.1371/journal.pcbi.1003334

**Published:** 2013-11-21

**Authors:** Luca Albergante, Jon Timmis, Lynette Beattie, Paul M. Kaye

**Affiliations:** 1 Department of Computer Science and Department of Electronics, University of York, York, United Kingdom; 2 Center for Immunology and Infection, Department of Biology and Hull York Medical School, University of York, York, United Kingdom; Ecole Normale Supérieure, France

## Abstract

Experimental visceral leishmaniasis, caused by infection of mice with the protozoan parasite *Leishmania donovani*, is characterized by focal accumulation of inflammatory cells in the liver, forming discrete “granulomas” within which the parasite is eventually eliminated. To shed new light on fundamental aspects of granuloma formation and function, we have developed an *in silico* Petri net model that simulates hepatic granuloma development throughout the course of infection. The model was extensively validated by comparison with data derived from experimental studies in mice, and the model robustness was assessed by a sensitivity analysis. The model recapitulated the progression of disease as seen during experimental infection and also faithfully predicted many of the changes in cellular composition seen within granulomas over time. By conducting *in silico* experiments, we have identified a previously unappreciated level of inter-granuloma diversity in terms of the development of anti-leishmanial activity. Furthermore, by simulating the impact of IL-10 gene deficiency in a variety of lymphocyte and myeloid cell populations, our data suggest a dominant local regulatory role for IL-10 produced by infected Kupffer cells at the core of the granuloma.

## Author Summary

Granulomatous inflammation is a common feature of chronic infectious and non-infectious disease. In the parasitic disease visceral leishmaniasis, the formation of granulomas in the liver is a hallmark of effective cellular immunity and host resistance to infection. Conventional experimental models, however, have inherent limitations in their capacity to assess the dynamics of this complex inflammatory response and in their ability to discriminate the local contribution of different immune cells and mediators to the outcome of infection. To overcome these limitations and to provide a future platform for evaluating how novel drugs might be used to improve host resistance, we have developed a computational model of the *Leishmania* granuloma. Using this model, we show that conventional measures of parasite load potentially mask an underlying heterogeneity in the ability of individual granulomas to control parasite number. In addition, we have used our model to provide novel insights into the relative importance of IL-10 production by different immune cells found within the granuloma microenvironment. Our model thus provides a complementary tool to increase understanding of granulomatous inflammation in this and other important human diseases.

## Introduction

Human visceral leishmaniasis (HVL or Kala azar) is the most severe form of the tropical disease leishmaniasis, and is caused by infection with the protozoan parasites *Leishmania donovani* or *L. infantum* [1]. HVL is a systemic disease, with the intracellular (amastigote) stage of the parasite found predominantly, but not exclusively, in mononuclear phagocytes of the spleen, bone marrow and liver of infected individuals. In the absence of treatment, HVL is usually fatal, with ∼40,000 deaths reported annually [2]. However, an estimated 90% of infections do not result in clinical disease. Evidence from epidemiological studies of HIV- *Leishmania* co- infection and from experimental studies indicate an important role for cellular immune mechanisms in controlling sub-clinical infection [3]. Evidence from studies in humans, from murine models of experimental visceral leishmaniasis (EVL) and from the study of canine visceral leishmaniasis (CVL) all point to an important role for T cell-derived cytokines in maintaining the balance of immunity during subclinical disease [4]. Furthermore, in each of these settings, there is evidence to suggest that granulomatous inflammation provides a histopathologic correlate of protective immunity [5]–[7].

The granuloma represents one of the defining tissue responses associated with chronic inflammation following a variety of microbial (e.g. *Schistosoma*, *Mycobacterium*, *Leishmania*) and non-infectious (e.g. autoimmune, prosthetics) insults. Although generally characterized by the focal accumulation of monocytes and T cells, recent data have indicated that a broad range of leucocytes can be found within these specialized microenvironments, including B cells, NK cells, NKT cells, T cells and dendritic cells. In addition, in some but not all cases, granulomas may progress to caseation, most commonly in tuberculosis, whereby neutrophils are seen in high abundance [8]–[12]. In EVL, granuloma formation has been shown to be dependent upon multiple cytokines, with the elimination of intracellular *Leishmania* governed by the balance of cytokines that are able to activate (e.g. IFNγ) or deactivate (e.g. IL-10) local macrophage anti-leishmanial activity [4], [5]. However, the relative functional contribution of different cell types producing similar cytokines, and whether these cells/cytokines exert their effects locally or indirectly (e.g. through upstream regulatory pathways operating outside the granuloma environment) remain as important but unanswered questions.

IL-10 is the best studied of the cytokines that have an inhibitory effect on macrophage leishmanicidal activity and serum IL-10 represents a biomarker of disease severity [13]. The current literature suggests multiple pathways in which IL-10 may operate [14], [15]. For example, expression of *Nos2* in macrophages, a key event in the generation of the leishmanicidal effector molecule nitric oxide, is directly inhibited by IL-10 [16]. Such inhibition may occur through autocrine signaling, with IL-10 being produced by macrophages after direct recognition of parasites or following immune complex binding to macrophage Fc receptors [17], [18]. Alternatively, IL-10 may indirectly regulate effector T cell differentiation and/or activation, e.g. by influencing the ability of macrophages and/or dendritic cells to stimulate T cell IFNγ production [19]. IL-10-producing DCs have been described in chronic EVL [20], [21] and CD4^+^ T cells which produce IL-10 (including natural Tregs, Tr-1 and CD4^+^ Th1 cells) have all been described in various forms of leishmaniasis in mouse and man [20], [22]–[28]. Given the potentially tissue damaging effects of uncontrolled inflammation, multiple cell populations within the granuloma may also develop self-regulating capacity, again with IL-10 as a component of this response. Thus, CD4^+^IFNγ^+^ Th1 cells and NK cells, which produce cytokines directing classical macrophage activation in the early stages of EVL, develop an IL-10-dependent immunoregulatory function as disease progresses [28], [29].

What has remained a considerable experimental challenge, however, has been to determine which of these cellular sources of IL-10 is functionally most potent in the local tissue microenvironment, which cells respond to the IL-10 signals and how this dynamic balance of immune effector and regulatory function evolves throughout the course of infection. In HVL, the invasive nature of the methods needed to address these questions is beyond what is practically or ethically achievable, and even in EVL, current methodology is still wanting. Nevertheless, there is an imperative to more fully understand these and related issues, if the information derived from past and future studies are to be effectively translated into new therapeutic approaches around this and other cytokines [30].

*In silico* models do not share the same experimental limits of *in vivo* models and allow more direct control on multiple experimental conditions. Computational and mathematical models of the cellular response to granulomatous infection have been developed previously in the context of tuberculosis [31]–[35], sarcoidosis [36] and leishmaniasis [37], but they generally account only for a limited number of leukocyte populations. For example, a recent study used a coloured Petri net approach to model the innate macrophage granuloma that forms during infection of zebrafish with *Mycobacterium* [38]. Here, we describe a stochastic Petri net model of granulomatous inflammation in the liver of mice infected with *L. donovani*, which faithfully reproduces many of the characteristics of experimental infection. Petri nets provide a visual-aided network-oriented modeling process, which simplifies their development and provides visual feedback affording interpretation by a broad audience. Moreover, the underlying mathematical structure of the model can be used to perform a structural validation, which is independent from the actual simulations. This validation assesses different properties of the model and can be used to detect entities that do not interact as intended or actions that will never be performed. These advantages have made stochastic Petri nets popular for the development of systems biology models [38], [39].

We have applied this methodology to gain a greater insight into the potential importance of macrophage deactivation in regulating the outcome of EVL, using this model to simulate the importance of macrophage deactivation mediated through IL-10. Our results indicate that local leishmanicidal activity is most strongly influenced through the action of IL-10 produced by infected Kupffer cells themselves rather than by infiltrating leucocytes. These results provide new insight into how effector mechanisms may be regulated within the granuloma, and a new tool to interpret how pharmacologic interventions may operate.

## Results/Discussion

### The essential features of granulomatous inflammation can be reproduced *in silico*

**Figure 1:**
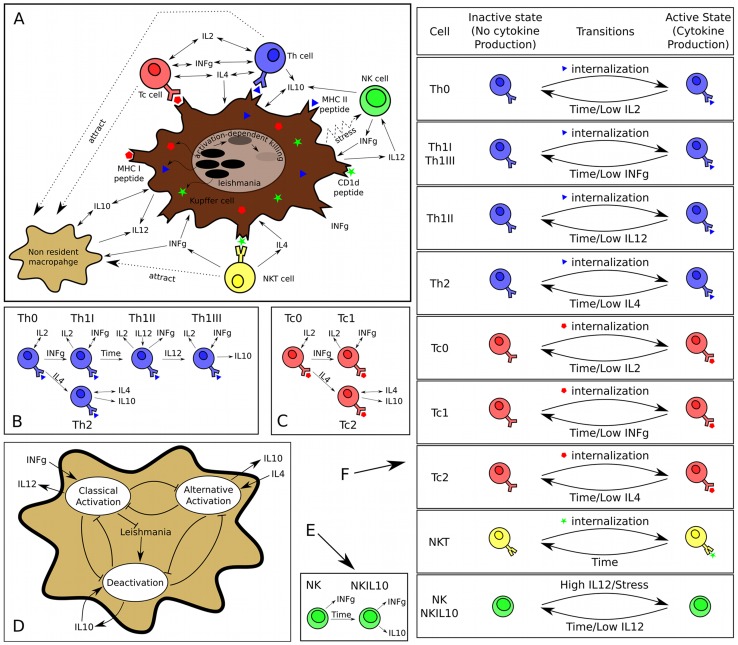
Schematics of the model dynamics. (**A**) High-level depiction of the interactions among the entities modeled. (**B**) Differentiation of helper T cells. Labels on arrows indicate the conditions for differentiation. Arrows pointing to/originating from a cytokine name indicate that the cytokine is produced/consumed by the cell. (**C**) Differentiation of cytotoxic T cells. Arrow conventions as in panel B. (**D**) Dynamics of activation types in macrophages. *Leishmania* interactions are restricted to Kupffer cells only. Note how different cytokines promote different types of activation and how different types of activation result in the production of different cytokines. (**E**) Differentiation of NK cells. Arrow conventions as in panel B. (**F**) Transitions from/to inactive to/from active states for the modeled leukocytes. This representation stresses the complexity of the model and the degree of interaction among the different cell populations; see Section 1 of Supplementary Information for a more detailed description.

Given the limited modeling work available in the context of EVL, we opted for a high-level population dynamics stochastic Petri net model that characterized many of the entities that are believed to be relevant for the progression of the disease. **Figure 1** presents a schematic representation of the model dynamics, and more detailed nets are described in **Figures S1, S2, S3, S4, S5, S6, S7 and Text S1**. The Petri net files are available for use in Snoopy (**Model S1**) and as a SBML compliant .xml file (**Model S2**). We chose to model granulomas as self-contained independently functioning units, i.e. without migration of cells or cytokine from one granuloma to another. Although we acknowledge that such events may occur, current experimental approaches in mice do not allow for these processes to be quantified. We defined a “baseline model” characterized by initial infection in resident Kupffer cells and a set of specific assumptions governing the behavior and inter-relationships between five cytokines (IL-2, IL-4, IL-10, IL-12 and IFNγ), a variety of effector/regulatory CD4^+^ and CD8^+^ T cell populations, NK and NKT cells and inflammatory monocytes/DC. **Text S2 and Tables S1, S2, S3, S4, S5, S6** describes the name, value and role of the different parameters used. Our baseline model does not include other populations of cells identified within granulomas but with less well-established roles in granuloma biology (e.g. B cells; [40]). Each cytokine was also associated with an ‘effectiveness’ parameter. This permits the same concentration of different cytokines to have different effects on cells, reflecting differences in specific activity, differences in sensitivity of signal transduction pathways and/or different numbers of receptors.

**Figure 2:**
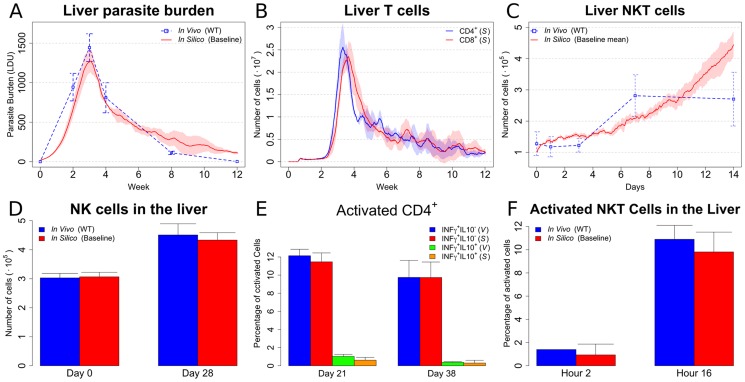
Baseline model reproduces many biological features of EVL. In all the panels, means and standard deviation are reported. Standard deviation is indicated by error bars or shaded areas (**A**) Organ level parasite burden (compared with [58]). (**B**) Number of CD4^+^ and CD8^+^ T cells over the course of infection of *in silico* data. The same plotting convention as panel A is used. (**C**) Number of NKT cells (compared with [59]). The same plotting conventions are used as in panel A. (**D**) **NK** cell number (compared with [29]). (**E**) Percentage of activated CD4^+^ T cells (compared with unpublished data). (V) and (S) indicate *in vivo* and *in silico*, respectively. (**F**) Percentage of activated NKT cells (compared with [59] and [60]).

As expected from the central limit theorem [41], the variability in simulated cell number depends on the number of simulations. Therefore, we performed experiments varying the number of independent granulomas. Although the infected liver contains an estimated 500,000 granulomas (based on counting the number of granulomas per unit volume in 2-photon 3D tissue images; data not shown), computational analyses suggested that 50 granulomas for each simulation were sufficient to generate a simulated total tissue parasite burden similar to that observed *in vivo* both in terms of mean and standard deviation (**Figure 2A**). Past studies on granulomatous inflammation have modeled granulomas as self-sustained microenvironments [34]. While different rationales for this approach exist, no clear biological evidence to support this view is available. Our data indicate that sampling a small subset of independent granulomas is sufficient to characterize the entire tissue response during infection. Although the qualitative variation of parasite burden over time reported by multiple investigators is similar, the absolute parasite burden may vary for a number of factors (e.g. parasite strain, animal husbandry, mouse strain etc.). Therefore, although to validate our model we choose a set of reference data [42], we compared the qualitative characteristics of the data only, and not the absolute quantitative values obtained. While this approach introduces simplifications over the *in vivo* situation, our results indicate that it provides a simple and manageable way to study the phenomenon of parasite killing within granulomas at an organ level.

To validate our model, we first ran simulations in which key parameters of immune function were measured. Our baseline model produced data that displayed good agreement with the published experimental data, in terms of the number of granuloma CD4^+^ and CD8^+^ T cells (**Figure 2B**); the number of NK cells (**Figure 2D**); the percentage of activated CD4^+^ T cells with differing functional activity (**Figure 2E**) and the frequency of activated NKT cells (**Figure 2F**). Nevertheless, the model did not predict all parameters of granuloma composition with strict accuracy. For example, there was a disparity in the absolute number of NKT cells within *in silico* granulomas compared to that observed in vivo (**Figure 2C**). This disagreement is likely due to biological constraints absent from our model, and indicates the importance of additional experimental work. Nevertheless, statistical analysis (**Text S3** and **Tables S7, S8, S9, S10, S11, S12, S13, S14**) supports the overall agreement between our simulations and experimental data points in the direction of robust modeling of the immune response.

### *In silico* modeling provides insight into the dynamics of leucocyte dynamics during infection

**Figure 3:**
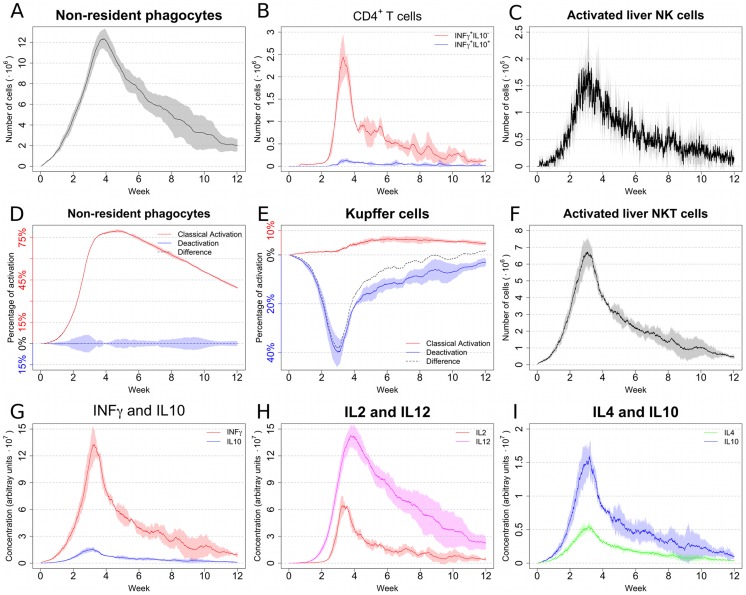
Baseline model allows the exploration of biological quantities difficult to access experimentally. In all the panels, means and standard deviation (indicated by shaded area around the mean) are reported. All numbers are relative to cells in the liver associated with a granuloma microenvironment. (**A**) Number of granuloma-associated non-resident macrophages. (**B**) Number of differentiated Th1 cells. (**C**) Number of activated NK cells. (**D**) Level of activation and deactivation of non-resident macrophages. (**E**) Level of activation and deactivation of Kupffer cells. (**F**) Number of activated NKT cells. (**G**) Concentration of IFNγ and IL-10. (**H**) Concentration of IL-2and IL-12. (**I**) Concentration of IL-4 and IL-10.

Since our *in silico* model allows a detailed characterization of the many biological entities involved in the immune response, we decided to explore their dynamics during infection. The number of non-resident phagocytes (**Figure 3A**), CD4^+^IFNγ^+^ T cells (**Figure 3B**), activated NK cells (**Figure 3C**), and activated NKT cells (**Figure 3F**) closely follow the dynamics observed for parasite burden. The number of CD4^+^IFNγ^+^IL-10^+^ T cells is very low when compared to CD4^+^IFNγ^+^IL-10^−^ T cells in the initial stages of infection, but their numbers become similar at later times (Figure 3B), perhaps suggesting a connection between IL-10-producing Th1 cells and the low level persistence of infection. A striking difference in kinetics is evident when comparing the activation status of non-resident phagocytes (**Figure 3D**) with resident Kupffer cells (**Figure 3E**). While in Kupffer cells the presence of parasites promotes a very high level of deactivation, non-resident phagocytes are strongly activated. This observation suggests that limiting the capacity of *Leishmania* to deactivate Kupffer cells could have a strong impact on the immune response. Moreover, while non-resident macrophages become strongly polarized, classical activation and deactivation coexists in Kupffer cells, resulting in a more dynamic and unstable equilibrium. Note that due to the very low rates of inflow and death, the number of Kupffer cells is effectively stable over time (data not shown).

Cytokines are effective only within a limited range. Unfortunately, accessing the concentration of cytokines in the granuloma microenvironment is experimentally beyond reach. Our data suggests that the concentration follows the parasite burden for all the modeled cytokines (**Figure 3G–I**). Notably, IL-10 concentration is much lower than IFNγ concentration over most of the time course of the infection (**Figure 3G**). As with the ratio of Th1 cells making IL-10 and those that do not (**Figure 3B**), the IFNγ: IL10 ratio decreases as peak parasite load is reduced and the rate of decay of liver parasite burden shallows off.

### Sensitivity analysis

The various entities included in the model were extensively parameterized, where possible using data extracted directly from the literature or from our own unpublished results. However, other parameters result from modeling decisions and simplifications and were determined by fitting the data. We therefore performed a sensitivity analysis to assess the robustness of the model. Here, we briefly describe the main points elucidated by sensitivity analysis. However, a more extensive discussion is provided in **Text S4**. Following established methodology [43], we sampled the parameter space using Latin hypercube sampling and studied the impact of each parameter on the parasite burden using Partial Rank Correlation Coefficients (PRCC). The effect of parameter variation was assessed on the parasite burden at different stages of the infection. As observed above, parasite burden correlates with most of the modeled aspects of the immune response. Hence, using parasite burden maximizes the information derived from the sensitivity analysis. Wherever possible, parameters were varied according to known biological variability. Additionally, given the stochastic nature of the model, a dummy parameter with no effect on the parasite burden was also included in the analysis. This expedient allowed us to disentangle the impact of the intrinsic variability of the model from the variability due to parameter variation.

As shown in **Figure S8**, varying model parameters has different impacts on parasite burden. This was to be expected given the differences in the number of leukocytes within and affecting the granuloma microenvironment. Varying the reproduction rate of the parasite, the effectiveness of IFNγ or the production of NKT-derived IFNγ resulted in a strongly varied parasite burden. However, varying the effectiveness of IL-2, the effectiveness of IL-10 and the chemokinetic effect of T cells did not significantly affect parasite burden. Sensitivity analysis confirmed the importance of many of the parameters believed to be the most important in determining the outcome of infection, again supporting the robustness of the model. However, less obvious results were also observed, and various parameters have remarkably different effects on parasite burden at different stages of the infection. (**Figures S9, S10, S11, S12, S13, S14, S15, S16**). This result stresses the complex dynamics that underlie the immune response during EVL and indicates how a cell with exactly the same behavior can potentially have very different degrees of biological significance at different stages of the infection.

### Individual granulomas display distinct dynamic behavior

**Figure 4:**
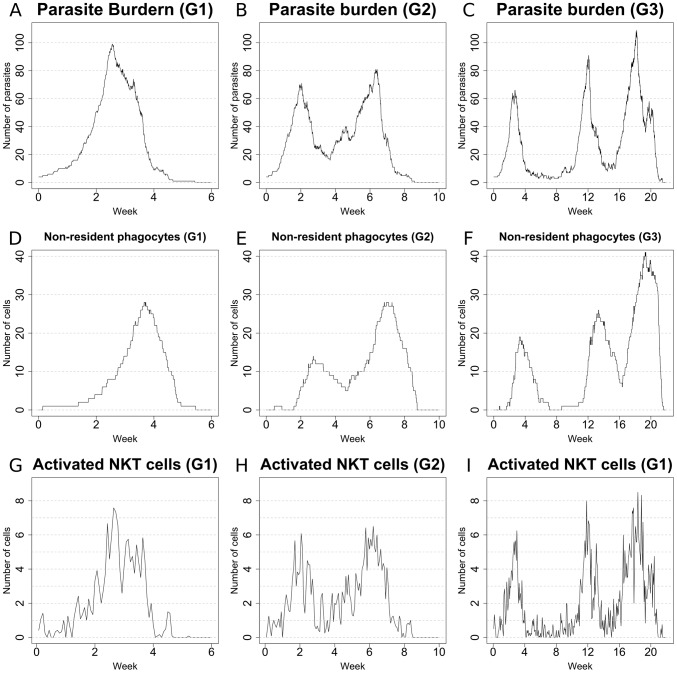
Sample behaviours of different *in silico* granulomas: 1. Number of parasites (**A**–**C**), number of non-resident phagocytes (**D**–**F**) and number of activated NKT cells (**G**–**I**) in sample granulomas. Note how remarkable diversity is observed among the different granulomas, with rather simple dynamics (**G1**) coexisting with more complex ones (**G2**, **G3**) in the simulations. Total organ parasite load can be reflected by the aggregate results from 50 granulomas.

Experimental data on parasite burden in mice is measured on a total organ basis (obtained from impression smears, limiting dilution analysis or quantitative PCR), with no experimental approaches being available to evaluate parasite number over time within individual granulomas. In contrast, our *in silico* model allows us to examine whether there is heterogeneity in parasite burden over time between individual granulomas. We observed that even under the same initial conditions (each granuloma seeded with 4 amastigotes), the peak parasite burden per granuloma varied greatly amongst individual granulomas (i.e. each represented by a single run of the simulation). More importantly, the dynamics of parasite burden displayed a remarkable variability between individual granulomas. Rather simple behaviors (granuloma G1: **Figure 4A**) coexisted with more complex ones (granuloma G2: **Figure 4B**). Notably, some granulomas (herein referred to as long-lasting) were characterized by an unexpected long-term equilibrium (granuloma G3; **Figure 4C**), with alternating high and low levels of parasite burden. Surprisingly, granuloma G1, though most effective at clearing parasites and maintaining effective control over recrudescence had the highest peak primary parasite load. This diversity in the parasite load is accompanied by a similar variation in the number of non-resident phagocytes (**Figures 4D–F**) and activated NKT cells (**Figures 4G–I**).

**Figure 5:**
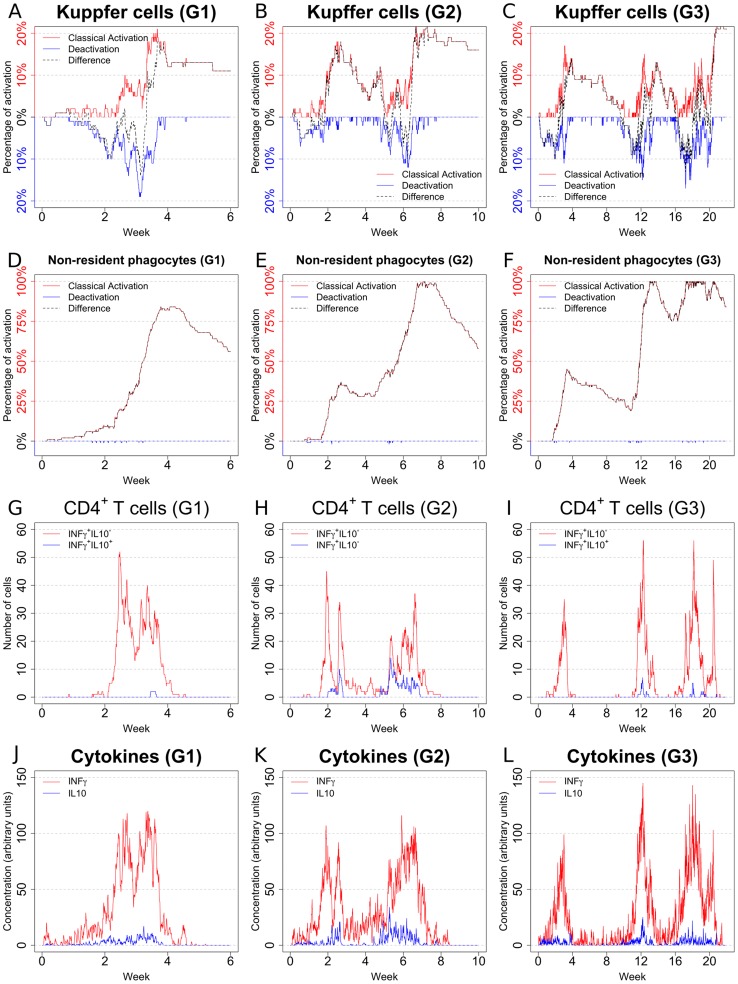
Sample behaviours of different *in silico* granulomas: 2. Level of activation in Kupffer cells (**A**–**C**; classical activation, red; deactivation, blue; difference, dotted), level of activation in non-resident phagocytes (**D**–**F**; classical activation, red; deactivation, blue; difference, dotted), number of activated effector Th1 cells (**G**–**I**; IFNγ^+^IL-10^−^, red; IFNγ^+^IL-10^+^, blue), and cytokine concentration (**J**–**L**; IFNγ, red; IL-10, blue) in samples granulomas.

To gain further insights into possible contingencies that can drive the emergence of the different types of granulomas observed, we explored the dynamics of the resident and non-resident phagocytes, the relations between the different populations of Th1 cells and the concentration of cytokines. The levels of classical activation and deactivation of Kupffer cells were comparable in all three granulomas. However, it was notable that the most effective granuloma (G1) was able to reach a relatively high level of activation (∼20%) and this was sustained past week 4 with negligible late deactivation (**Figure 5A**), whereas granulomas G2 and G3 took more time to reach maximal classical activation and the degree of classical KC activation fell more sharply after the primary peak of parasite load concomitant with a degree of deactivation (**Figures 5B and 5C**). The activation of non-resident macrophages follows a similar trend with a high level of activation reached in granuloma G1 by week 4 (**Figure 5D**), while granulomas G2 and G3 again did not reach the same level of classical activation in response to the primary peak of parasite load (**Figures 5E and 5F**). The number of IFNγ^+^IL-10^−^ T cells was comparable in all 3 granulomas considered (**Figures 5G–H**). However, IFNγ^+^IL10^+^ T cells were practically absent in granuloma G1 (**Figure 5G**), while these cells were present in moderately high numbers in granulomas G2 and G3 (**Figures 5H and 5I**). These findings further support a role for IFNγ^+^IL10^+^ T cells in the persistence of infection. Nevertheless, IFNγ^+^IL10^+^ T cells appear quite late in G3, suggesting that they are not the only factor contributing to delayed clearance. Finally, the cytokine concentration is related to the number of Th1 cells (**Figures 5J–L**), supporting the importance of T cells in the control of the local environment.

From a biological perspective, our results provide the first demonstration that local immune control within granulomas may be variable, with important implications for how the effector response is regulated. In particular, these results suggest that the local environment within the granuloma shapes the effector response and that granulomas may behave as autonomous units of immune function. Conceptually, this model of autonomous units of immunity is supported by recent data that suggests local antigen-specific re-stimulation is required for T cell effector function [44]. Additionally, our model suggests that a limited number of long-lasting granulomas, which would be difficult to detect with standard parasite counting techniques, may provide a reservoir for disease reactivation in murine models of T cell insufficiency [45] and this has implications for the immunological basis of long-term parasite persistence that warrant further exploration. Although we have not extensively investigated this point to date, the small sample of granulomas sampled suggests that those with highest initial peak parasite burden may actually be the most effective at controlling parasite load over the long term.

From a modeling perspective, the diversity of parasite loads seen in individual granulomas obtained in the simulations supports the power of stochastic models: the same Petri net is able to generate widely diverse parasite numbers in different granulomas over time. However, averaging these burdens leads to a quite characteristic (and mostly deterministic) “shape” for graphs of parasite burden determined at an organ level. The emergence of a definite trend from stochastic interactions is not a new theme, and is a founding principle of statistical thermodynamics. However, the emergence of multi-scale behaviors (stochastic for the microenvironment and deterministic at the organ level) indicates the importance of multi-level studies when trying to understand biological processes.

### The impact of *in silico* immunodeficiency on the course of infection

**Figure 6:**
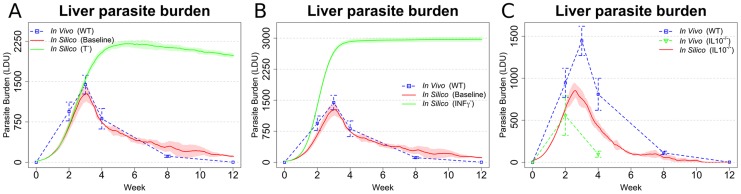
Simulations reflecting gene KO qualitatively reproduce expected changes in disease outcome. (**A** and **B**) Parasite burden after *in silico* knock out of T cells (A) or IFNγ (B), compared to the results from the baseline model (baseline) and in vivo ([58]; WT). (**C**) *In silico* knock out of IL-10 compared with [58] and data adapted from [61]). In all the panels, means and standard deviation are reported. Standard deviation is indicated by error bars or shaded areas.

Having established that our model recapitulated many of the features of infection seen in immunocompetent mice, we then went on to explore how well we could recapitulate the differing outcomes of infection as seen in cell or cytokine deficient mice. T cells are considered to be the main mediators of cellular immunity against *Leishmania* and not surprisingly, depletion of T cells in our *in silico* model (**Figure 6A**) led to high parasite burden and a non-healing course of infection, reflecting the course of infection in T cell-deficient mice [5]. The cytokine IFNγ is considered fundamental for the classical activation of macrophages [46] and mice deficient in IFNγ are unable to mount an adequate immune response and are highly susceptible to EVL [5]. Likewise in our model, inhibiting production of IFNγ from all cells capable of producing this cytokine lead to very high parasite burden and to a non-healing infection (**Figure 6B**). In contrast to IFNγ, IL-10 has a negative impact on host resistance, with IL-10 KO mice or mice treated with anti-IL-10R having enhanced resistance to infection [47], [48]. Our model also indicated an important role for IL-10 in regulating parasite burden, as blocking the production of IL-10 by all leukocytes resulted in a reduced peak parasite burden (**Figure 6C**), though to a slightly lesser extent compared to that reported in IL-10 KO mice. Overall, however, the model appeared to faithfully recapitulate many of the key features of EVL observed in various immunodeficient mouse models.

### KC-derived IL-10 plays a dominant role in regulating granuloma effector function

**Figure 7:**
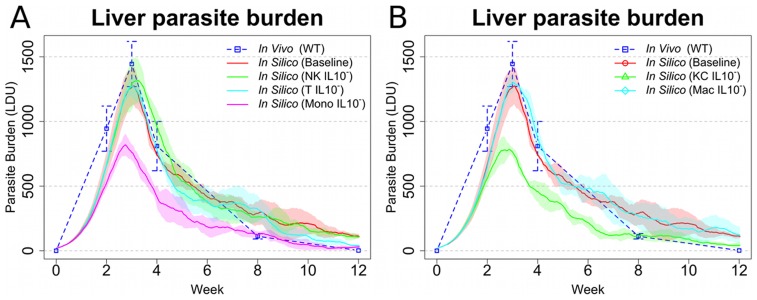
*In silico* cell-specific knock out of IL-10 implicates Kupffer cell IL-10 production as a major determinant of leishmanicidal activity within granulomas. (**A**) *In silico* knockout of IL-10 in mononuclear phagocytes (Mono IL-10^−^), T cells (T IL10^−^), and NK cells (NK IL10^−^) compared with baseline *in silico* model and in vivo ([58]; WT). (**B**) *In silico* knockout of IL-10 from Kupffer cells (KC IL10^−^) and non-resident macrophages/monocytes/DC (Mac IL10^−^), compared with baseline *in silico* model and [58]. In all the panels, means and standard deviation are reported. Standard deviation is indicated by error bars or shaded areas.

In EVL, multiple cellular sources of IL-10 have been described, including IL-10-producing CD4^+^ Th1 cells [20], NK cells [29], and inflammatory macrophages (monocytes and DCs [20]). Kupffer cells are also well known to produce IL-10 under differing conditions [49], [50]. On the basis that our model was able to demonstrate the effects of global IL-10 deficiency (**Figure 6C**), we proceeded to conduct a series of predictive studies to identify which cell(s) produced IL-10 with most impact on the effector function of the granuloma (in essence generating a series of conditional cell-specific IL-10 gene deficiencies *in silico*). Our simulation results indicated that removing the capacity of CD4^+^ IFNγ^+^ Th1 cells, inflammatory monocytes/DC, and IFNγ^+^ NK cells to make IL-10 all had perceivable but different impacts on parasite burden (**Figure 7A**). Removing the capacity of CD4^+^ IFNγ^+^ Th1 and NK cells to produce IL-10 did not alter the peak parasite burden, but delayed final stages of parasite clearance. In contrast, removing the capacity of myeloid cells (including KCs and inflammatory DC/monocytes) to produce IL-10 had a much more significant impact on parasite burden evident by a reduction in peak tissue parasite load and a more rapid resolution of infection. Qualitatively, the effect of IL-10 depletion from myeloid cells alone was similar to that of global IL-10 depletion (**Figure 6C**).

To further dissect the role of different myeloid cells and the relative contribution of autocrine IL-10 production by infected KC and IL-10 that was derived from inflammatory monocytes/DC, we selectively eliminated IL-10 production by each population. Our results clearly indicated that IL-10 production by KCs had a greater impact on parasite burden compared to IL-10 production by inflammatory monocytes/DC (**Figure 7B**). Indeed, our model predicts the outcome of KC IL-10 deficiency to be as effective in increasing host resistance as total ablation of IL-10 (**Figures 6C** **and** **7B**). Thus, while supporting a role for IFNγ^+^IL10^+^ T cells and IL-10^+^ NK cells in the regulation of immunity to *L. donovani*, our data suggests that IL-10 production by infected KCs themselves plays the most dominant role in regulating intra-granuloma effector function. This finding is clearly a consequence of the high level of deactivation observed in Kupffer cells at both global (**Figure 3E**) and local scales (**Figures 5A–C**), which promotes IL-10 production by these cells.

### Concluding remarks

*In silico* models are increasingly important tools to elucidate complex biological phenomena. However, selecting the adequate level of abstraction and controlling its complexity can be daunting tasks when studying the diverse biological mechanisms used by the vertebrate immune system. Care is needed in building *in silico* models with clear working hypotheses and extensive validation. In comparison to other *in silico* models of granuloma formation, our model provides a systematic view of the course of infection, without sacrificing the complexity and diversity of the immune response. Our model of course contains a number of explicit simplifications and assumptions, and whilst based on the literature, many of these may be open to alternate interpretation or indeed not operate as hard rules. However, an extensive list of the working hypotheses and an assessment of the effect of the different model parameters by sensitivity analysis (**Text S4**) provides further insight into the working of the model and its robustness. In contrast, a disadvantage of our systemic view is that unlike agent based models [51], Petri net models are not able to elucidate the role played by physical interactions among leucocytes within a spatially defined environment. Additionally, in comparison to simpler deterministic models, our analyses are performed only by means of simulations, thus limiting the exploration of the parameter space.

Notwithstanding these limitations, our current model captures many experimentally determined features of the *Leishmania* granuloma and has provided a number of unique and important insights into the aspects of granuloma function that are currently intractable in vivo. First, our observation that individual granulomas display distinct patterns of effector function over time suggests a novel means for the maintenance of persistent parasites in tissues and has implications for understanding parasite recrudescence. Second, the prediction from our model that autocrine IL-10 production by KCs is a major regulator of leishmanicidal activity within granulomas was unexpected, given the recent attention focused on the production of this cytokine by T cells and NK cells, including from our own work. Importantly, it should be noted that our model only simulates events within the granuloma that lead to the control of parasite load and hence does not take into account the possible regulatory effects of IL-10 outside this environment. For example, our model would not capture possible effects of IL-10 on T cell differentiation occurring in other tissues, but which might affect the quality and/or quantity of T cells that enter into the granuloma microenvironment.

Our model also adds to a growing body of modeling literature on macrophage activation. Macrophages have the capacity to assume different states of functional ‘activation’ dependent in part upon the cytokine environment to which they are exposed (variously defined as M1 and M2 or as classical and alternate activation, respectively) with profound implications for the control of intracellular pathogens and the regulation of inflammation (reviewed in [52], [53]). The interplay between classical and alternate activation has been previously modeled in the context of tuberculosis [33], [54], [55]. Alternatively activated macrophages (driven by IL-4) are limiting in our model, whereas de-activation resulting from non-cytokine as well as cytokine mediated mechanisms acts as the principal counter balance to classical activation. Nevertheless, taken together these studies are providing novel insights into how macrophage effector function is regulated in a dynamic way over the course of infection and illustrate how changes in the balance of activation phenotypes at the local site can shape the outcome of infection. It remains to be determined whether such information can be harnessed to develop useful and clinically relevant prognostic indicators of disease outcome.

In conclusion, this novel stochastic Petri net model of granuloma formation provides a new tool to complement other approaches to understand the biology of granulomatous inflammation and the determinants of host resistance to *L. donovani*. Whilst our *in silico* studies indicate the potential for enhancing local immunity through disabling IL-10 production in a cell-specific manner, final validation of the predictions made by this model will require the future development of novel KC-specific IL-10 deficient mice.

## Materials and Methods

### Animal experiments

Data on the cellular composition of the *Leishmania* granuloma in mice, collected by real time intravital imaging and the analysis of disaggregated liver tissue, was used for parameterization of the model. Data from the authors' laboratory were collected under license from the UK Home Office (PPL 60/4377). All animal experiments were approved by the University of York Ethical Review Process and studies were conducted in accord with ARRIVE guidelines and the principles of the 3Rs.

### Formalism and tools

The model used was developed using stochastic Petri nets and simulated with Snoopy [56]. For each *in silico* experiment, the populations of entities at the organ level were calculated using the averages of 50 independently simulated granulomas and by multiplying these averages by the calculated number of granulomas in the entire liver (5×10^5^). *In silico* values displayed in the figures were obtained by averaging the results of 3 experiments under the same conditions, the error bars indicate sample standard deviation. Leishman Donovan Units (LDU) values were obtained by dividing the number of parasites by 10^5^ [57].

### Dynamics of modeled entities

The number of individuals for any given population was modeled by the number of tokens in the place representing that population. Besides some transitions modeling the time-flow, the firing time for a transition was modeled by a negative exponential distribution. The following entities were considered by the model: Cytokines (IL-2, IL-4, IL-10, IL-12, and IFNγ), mononuclear phagocytes (Kupffer Cells, non-resident macrophages/monocytes/DC), *L. donovani*-derived peptides associating with MHC and non-classical MHC molecules, NK cells, NKT cells, and CD8^+^ and CD4^+^ T cells. A detailed description of the dynamics of the above entities is provided in **Text S2**.

### Statistical analysis

Statistical analysis was performed on the mean using Student's t-test with Welch's correction for the degrees of freedom. P-value was calculated for equality test (see **Text S3** for further details).

### Sensitivity analysis

Both Latin hypercube sampling and partial rank correlation coefficients are widely used techniques. A formal presentation of these techniques can be found in [43]. Briefly, PRCC characterizes the strength of the linear relationship between two variables after a correction has been made for the linear effects of the other variables considered by the analysis. Additionally, p-value indicates the probability of getting a correlation as large as the observed value by random chance, when the true correlation is zero. P-values lower than 0.05 (or 0.01) were considered indicative of statistical significance. See **Text S4** for further details.

## Supporting Information

Figure S1 **High Petri nets.** This net indicates the interactions among the entities of the model. Detailed nets for the single coarse place are depicted in **Figures S2 to S7**.
(TIFF)

Figure S2 **Environmental cytokines.** Some places represent the concentration of cytokines and the transitions model processes that produce or consume them. Additional places are used to block the production of certain cytokines.
(TIFF)

Figure S3 ***Leishmania*** **parasites.** The places represent the number of parasites. The transitions model the processes that are affected by their number, their killing and their reproduction.
(TIFF)

Figure S4 **Phagocytes** The places represent the number of phagocytes, their state, and the concentration of MHC peptides. The transitions model their migration, death, emigration, and state-change. Non-resident macrophages (top) and KCs (bottom).
(TIFF)

Figure S5 **Natural Killer cells.** Some places represent the number of NK cells. The transitions model the activation, migration and emigration. One place is used as marker to remove NK cells from the model.
(TIFF)

Figure S6 **Natural Killer T cells.** Some places represent the number of NKT cells. The transitions model the activation, migration and emigration. One place is used as a marker to remove NKT cells from the model.
(TIFF)

Figure S7 **T cells.** Some places represent the number of T cells. The transitions model the activation, migration, emigration and death. One place is used as marker to remove T cells from the model.
(TIFF)

Figure S8 **Sensitivity analysis of the parameters of the model.** The PRCC at various times is reported. When the value is statistically significant in at least one of the time chosen, a sequence of ‘*’ and ‘-’ is depicted. ‘*’ represents statistically significant (p-val<0.05), while ‘-’ represents non-statistically significant, and the sequence indicated the statistical significance at 100, 500, and 1000 hours.
(TIFF)

Figure S9 **PRCC of LDRep. Effect of the variation of the fixed reproduction of the parasite on the organ level parasite burden.** The positive values between hour 0 and hour 500 indicate that an increase of reproduction rate results in a higher parasite burden. The positive values after hour 500 indicate that an increase of reproduction rate results in a lower parasite burden. Dotted line indicates the effect due to model stochasticity. Shaded area indicates statistically significant results. Taken together, these results indicate that a faster reproduction favors the parasite in the short run, but reduce the probability of a chronic infection.
(TIF)

Figure S10 **The reciprocal down-regulation of (de)activation is important in the final stages of EVL.** The PRCC of ActivationFight indicates that a faster down-regulation (and thus a higher value of the parameters) leads to a reduced parasite burden in the final stages of the infection.
(TIF)

Figure S11 **The production of CD1d peptides is important in controlling the parasite burden in the early stages of EVL.** The PRCC of CD1dProd indicates that an increase in the amount of peptides displayed by KCs (and thus a higher value of the parameters) leads to a reduced parasite burden in early stage of infection, supporting the important role of NKT cells in the initial immune response to EVL.
(TIF)

Figure S12 **The cytokine production of T and NKT cells is important in controlling the parasite burden.** The PRCC of TCellCytProd (top) and iNKTIFN*γ*Prod (bottom) indicates that an increase in the amount of cytokine produced by T cell or in the amount of IFN*γ* produced by NKT cells (and thus a higher value of the parameters) leads to a reduced parasite burden, supporting the important role of T and NKT cell-derived cytokines.
(TIF)

Figure S13 **A faster diffusion of cells or cytokine has a positive impact on the parasite burden.** The PRCC of CellDiff (top) and CytDiff (bottom) indicate that a faster diffusion of the cells or cytokine results in a larger parasite burden.
(TIF)

Figure S14 **A stronger effectiveness of cytokines has the expected impact.** The PRCC of IL-10Effectiveness (top) and IFN*γ*Effectiveness (bottom) indicate that the cytokines behave as expected. A more effective IFN*γ* results in a lower parasite burden, while a more effective IL-10 results in a high parasite burden.
(TIF)

Figure S15 **More stable MHC complexes reduce the parasite burden.** The PRCC of MHCILife indicates that a longer half-life of the complexes leads to a lower parasite burden. This result is compatible to the stronger T cell response that more stable MHC-peptide complexes are likely to elicit.
(TIF)

Figure S16 **The activation and deactivation of T cells strongly impact the parasite burden.** The PRCC of TCellAct (top) and TDeact (bottom) confirm the important role of T cells in the immune response to EVL. A larger probability of activation, respectively deactivation, results in a lower, respectively larger, parasite burden as expected.
(TIF)

Model S1 **Petri net file for use in Snoopy editor.**
(ZIP)

Model S2 **Validated Systems Biology Markup File (SBML) file.**
(XML)

Table S1 **Environment-related parameters.**
(DOCX)

Table S2 ***Leishmania*** **-related parameters.**
(DOCX)

Table S3 **Macrophage-related parameters.**
(DOCX)

Table S4 **NKT cell-related parameters.**
(DOCX)

Table S5 **NK cell-related parameters.**
(DOCX)

Table S6 **T cell-related parameters.**
(DOCX)

Table S7 **P-values for LDU means equality in** [**13**] **and scaled** [**12**] **.**
(DOCX)

Table S8 **P-values for LDU means equality in** [**13**] **and scaled** [**11**] **.**
(DOCX)

Table S9 **P-values for LDU means equality** ***in vivo*** **and** ***in silico*** **.**
(DOCX)

Table S10 **P-values for NKT cells number means equality** ***in vivo*** **and** ***in silico*** **.**
(DOCX)

Table S11 **P-values for percentage of IFN** ***γ^+^*** **IL-10^−^ cells means equality** ***in vivo*** **and** ***in silico*** **.**
(DOCX)

Table S12 **P-values for percentage of IFN** ***γ^+^*** **IL-10^+^ T cells means equality** ***in vivo*** **and** ***in silico*** **.**
(DOCX)

Table S13 **P-values for number of NK cells means equality** ***in vivo*** **and** ***in silico*** **.**
(DOCX)

Table S14 **P-values for percentage of activated NKT cells means equality** ***in vivo*** **and** ***in silico*** **.**
(DOCX)

Table S15 **Percentage of helper T cells.**
(DOCX)

Text S1 **Petri Nets.**
(DOCX)

Text S2 **Dynamics of modeled entities.**
(DOCX)

Text S3 **Statistical analysis.**
(DOCX)

Text S4 **Sensitivity analysis.**
(DOCX)

Text S5 **Assumptions and simplifications of the model.**
(DOCX)

Text S6 **Supplementary references.**
(DOCX)
