# Supplementary figures and images for: A Petri Net Model of Granulomatous Inflammation: Implications for IL-10 Mediated Control of Leishmania donovani Infection
Source: PLoS Comput Biol. 2013 Nov 21;9(11):e1003334. doi: 10.1371/journal.pcbi.1003334 (PMC3867212; doi:10.1371/journal.pcbi.1003334)

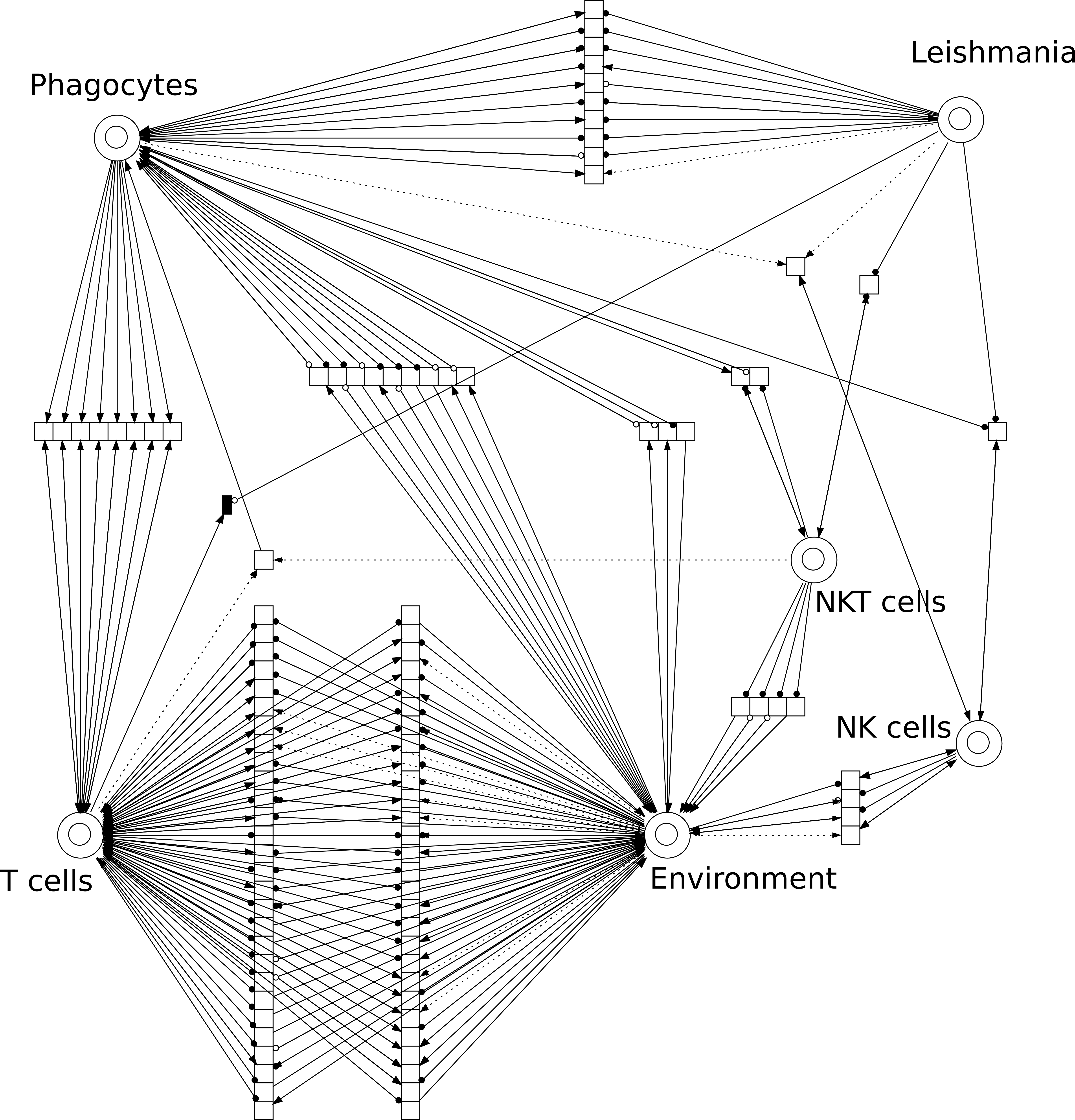

Supplement: Figure S1 — High Petri nets. This net indicates the interactions among the entities of the model. Detailed nets for the single coarse place are depicted in Figures S2 to S7. (TIFF) [file pcbi.1003334.s001.tiff]

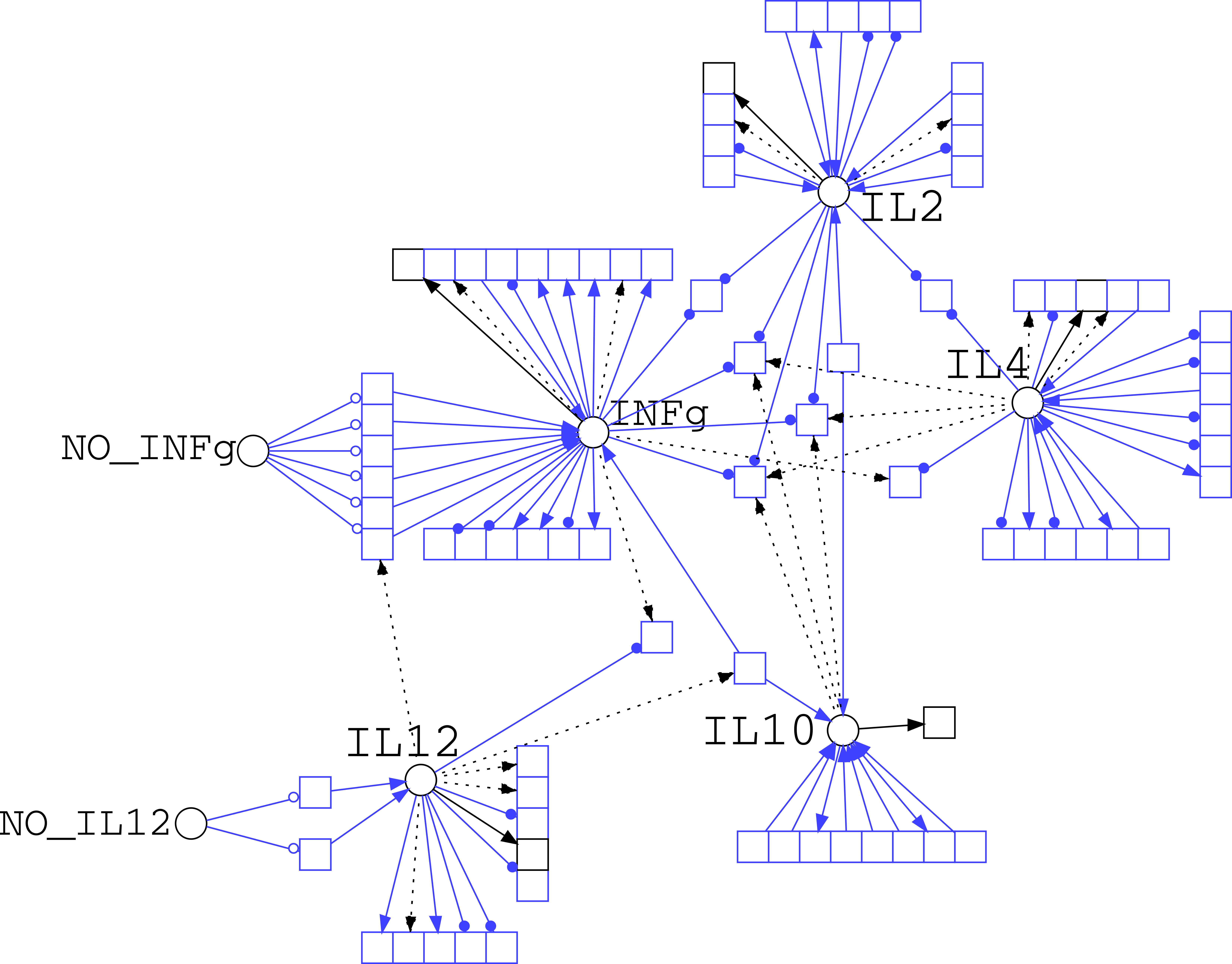

Supplement: Figure S2 — Environmental cytokines. Some places represent the concentration of cytokines and the transitions model processes that produce or consume them. Additional places are used to block the production of certain cytokines. (TIFF) [file pcbi.1003334.s002.tiff]

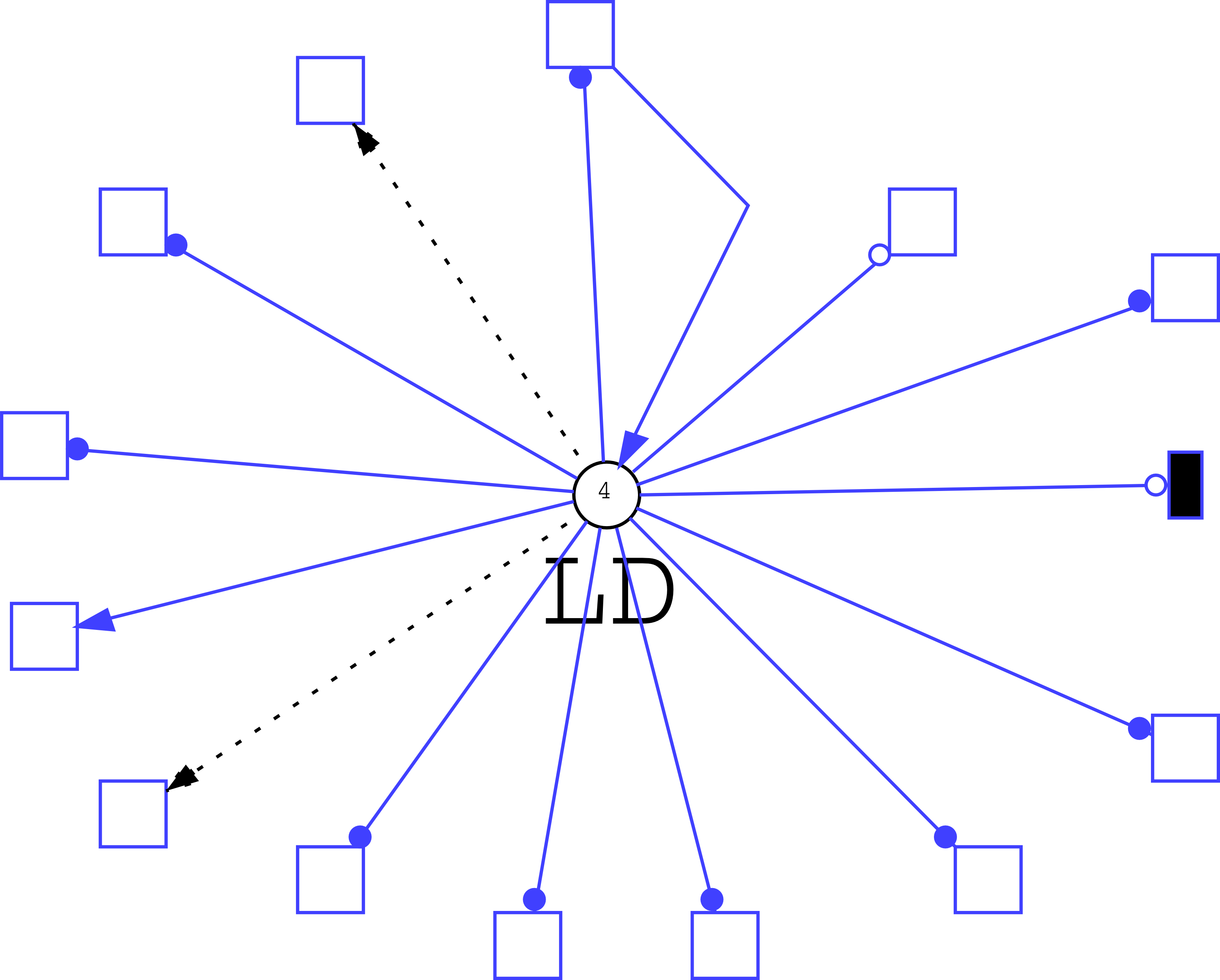

Supplement: Figure S3 — Leishmania parasites. The places represent the number of parasites. The transitions model the processes that are affected by their number, their killing and their reproduction. (TIFF) [file pcbi.1003334.s003.tiff]

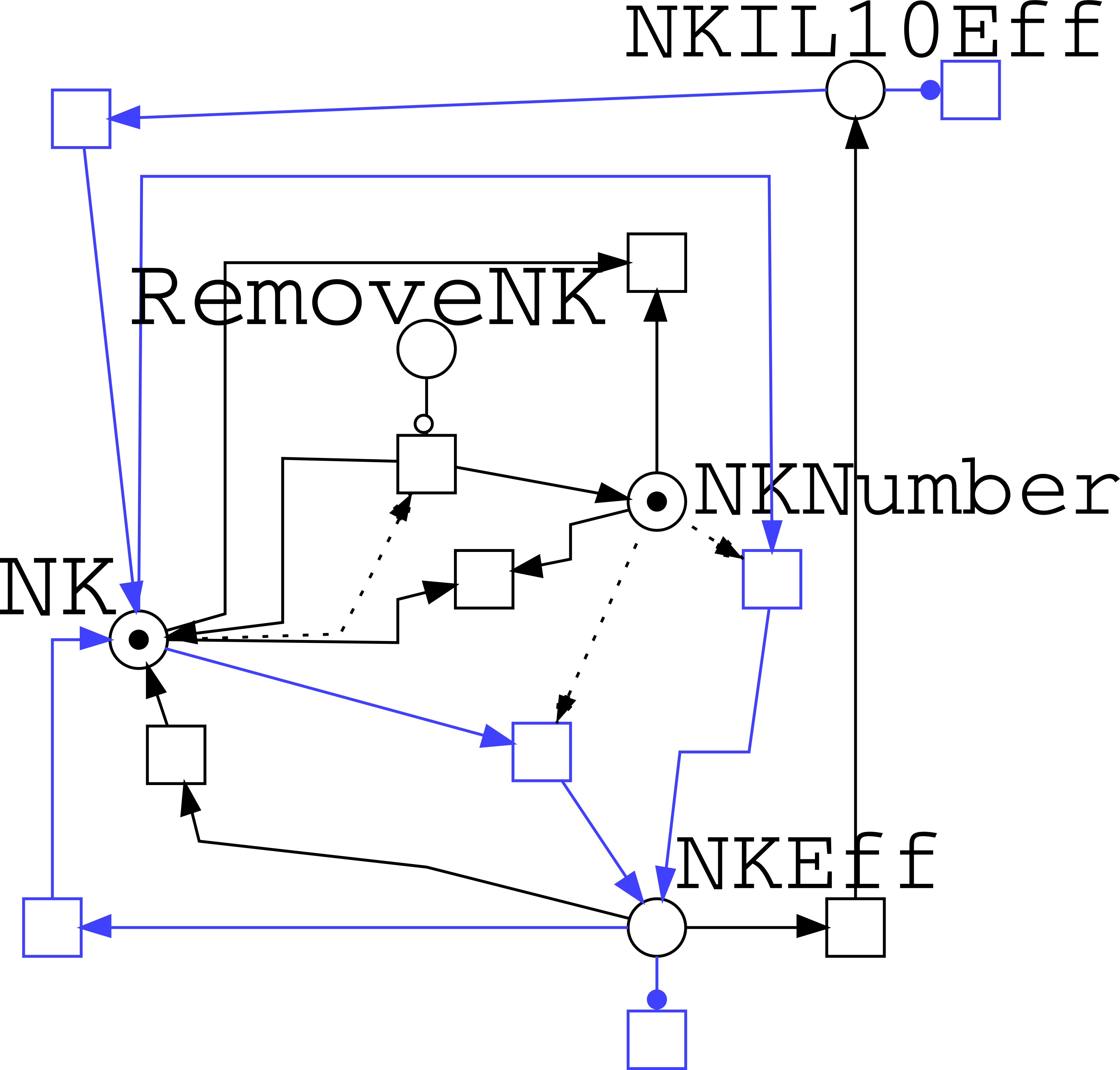

Supplement: Figure S5 — Natural Killer cells. Some places represent the number of NK cells. The transitions model the activation, migration and emigration. One place is used as marker to remove NK cells from the model. (TIFF) [file pcbi.1003334.s005.tiff]

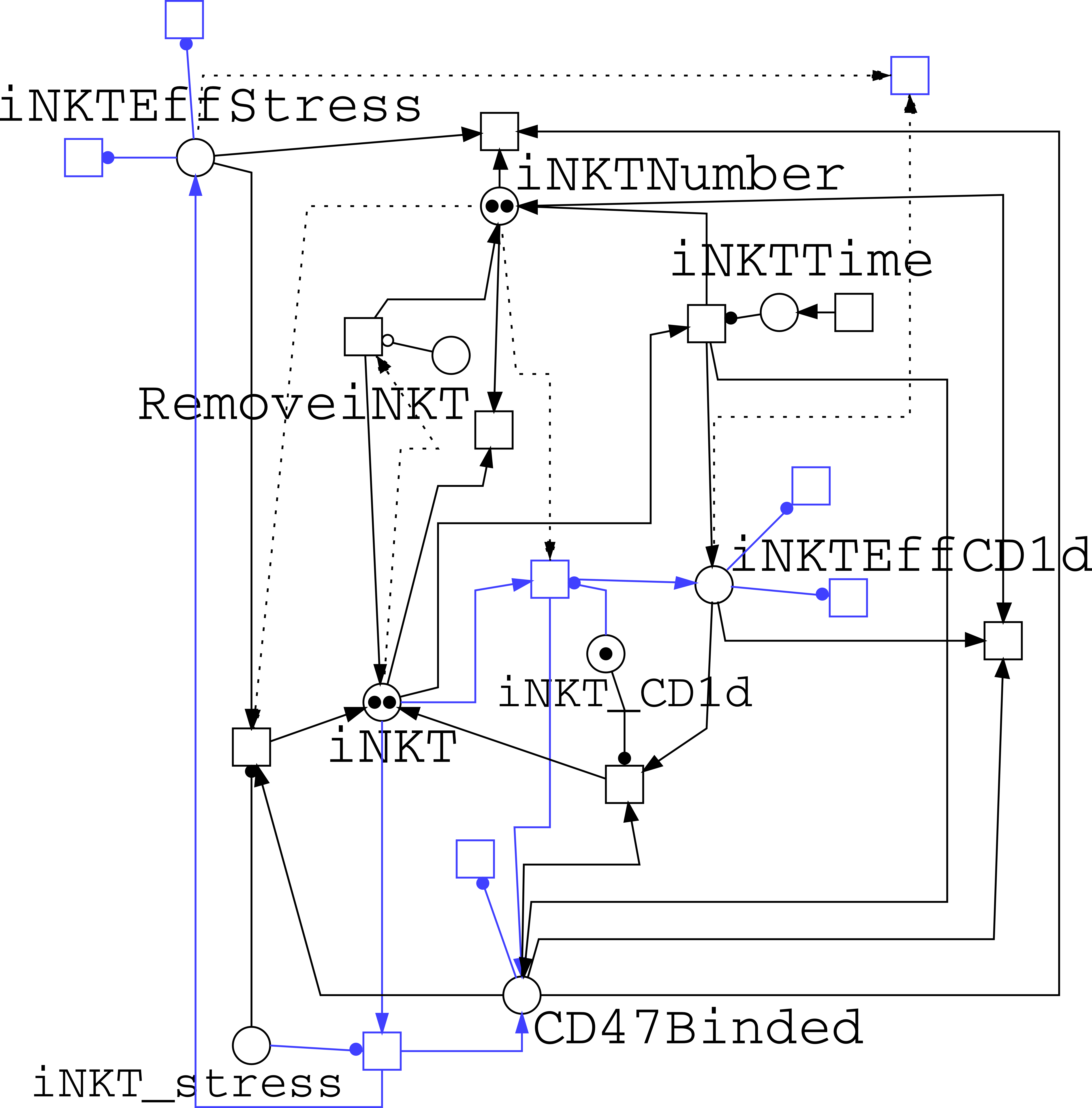

Supplement: Figure S6 — Natural Killer T cells. Some places represent the number of NKT cells. The transitions model the activation, migration and emigration. One place is used as a marker to remove NKT cells from the model. (TIFF) [file pcbi.1003334.s006.tiff]

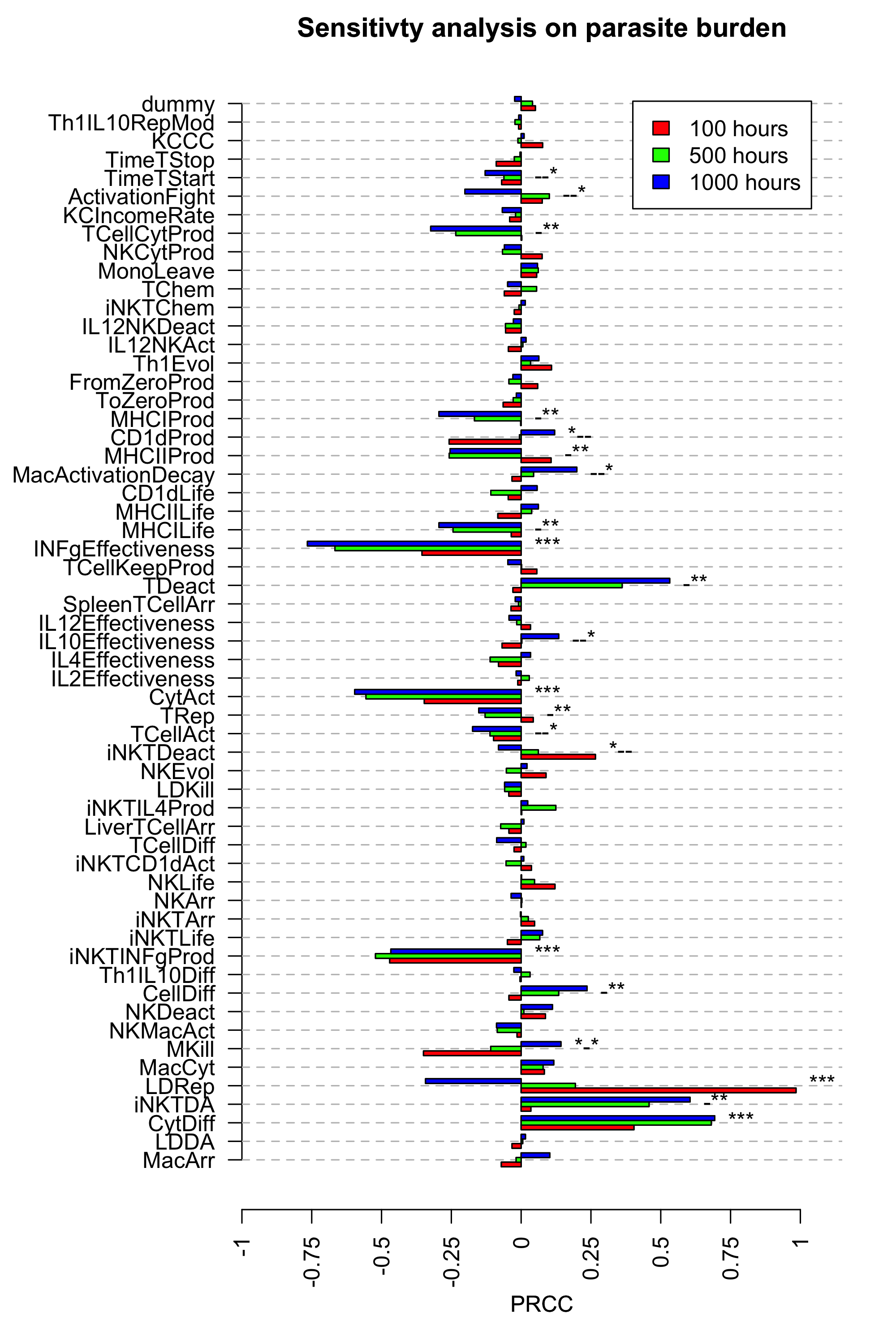

Supplement: Figure S8 — Sensitivity analysis of the parameters of the model. The PRCC at various times is reported. When the value is statistically significant in at least one of the time chosen, a sequence of ‘*’ and ‘-’ is depicted. ‘*’ represents statistically significant (p-val<0.05), while ‘-’ represents non-statistically significant, and the sequence indicated the statistical significance at 100, 500, and 1000 hours. (TIFF) [file pcbi.1003334.s008.tiff]
