# Supplementary material for: A Petri Net Model of Granulomatous Inflammation: Implications for IL-10 Mediated Control of Leishmania donovani Infection
Source: PLoS Comput Biol. 2013 Nov 21;9(11):e1003334. doi: 10.1371/journal.pcbi.1003334 (PMC3867212; doi:10.1371/journal.pcbi.1003334)
Supplement: Table S1 — Environment-related parameters. (DOCX) [file pcbi.1003334.s019.docx]

| **Parameter** | **Value** | **Description** |
| --- | --- | --- |
| CytDiff | 0.25 | controls the exponential decay of cytokines due to blood flow |
| CellDiff | 0.05 | controls the exponential decay of homeostatic cells due to blood flow |
| IL-2Effectiveness | 0.1 | controls the effectiveness of IL-2 |
| IL-4Effectiveness | 0.8 | controls the effectiveness of IL-4 |
| IL-10Effectiveness | 1.3 | controls the effectiveness of IL-10 |
| IL-12Effectiveness | 1 | controls the effectiveness of IL-12 |
| IFN*γ*Effectiveness | 0.8 | controls the effectiveness of IFN*γ* |
