# Supplementary material for: A Petri Net Model of Granulomatous Inflammation: Implications for IL-10 Mediated Control of Leishmania donovani Infection
Source: PLoS Comput Biol. 2013 Nov 21;9(11):e1003334. doi: 10.1371/journal.pcbi.1003334 (PMC3867212; doi:10.1371/journal.pcbi.1003334)
Supplement: Table S2 — Leishmania -related parameters. (DOCX) [file pcbi.1003334.s020.docx]

| **Parameter** | **Value** | **Description** |
| --- | --- | --- |
| LDKill | 0.00001 | controls the ability of the parasites to kill the KCs that ingested them |
| LDRep | 0.015 | controls the reproduction of the parasites |
| LDDA | 0.0001 | controls the deactivation capacity of the parasite on the infected KCs |
