# Supplementary material for: A Petri Net Model of Granulomatous Inflammation: Implications for IL-10 Mediated Control of Leishmania donovani Infection
Source: PLoS Comput Biol. 2013 Nov 21;9(11):e1003334. doi: 10.1371/journal.pcbi.1003334 (PMC3867212; doi:10.1371/journal.pcbi.1003334)
Supplement: Table S3 — Macrophage-related parameters. (DOCX) [file pcbi.1003334.s021.docx]

| **Parameter** | **Value** | **Description** |
| --- | --- | --- |
| CytAct | 0.005 | controls the sensitivity of macrophages to cytokines |
| MKill | 0.09 | controls the ability of KCs to kill ingested parasites |
| MHCIProd | 0.079 | controls the production of MHC class I peptides by KC |
| MHCIIProd | 0.0163 | controls the production of MHC class II peptides by KC |
| CD1dProd | 0.0179 | controls the production of CD1d peptides by KC |
| MHCILife | 3 (from[3]) | half-life of MHC class I peptides |
| MHCIILife | 60 (from [4]) | half-life of MHC class II peptides |
| CD1dLife | 20 (from [5] ) | half-life of CD1d peptides |
| MacArr | 0.4 | controls the arrival of non-resident macrophages |
| MonoLeave | 0.15 | controls the departure of non-resident macrophages |
| KCIncomeRate | 0.0001 | controls the inflow of KCs |
| MacCyt | 3 | controls the cytokine production of macrophages |
| ActivationFight | 0.05 | controls the pairwise down-regulation of (de)activations |
| MacActivationDecay | 0.001 | controls the time-dependent decay of (de)activations |
| KCCC | 100 | “carrying capacity” of KC |
