# Supplementary material for: A Petri Net Model of Granulomatous Inflammation: Implications for IL-10 Mediated Control of Leishmania donovani Infection
Source: PLoS Comput Biol. 2013 Nov 21;9(11):e1003334. doi: 10.1371/journal.pcbi.1003334 (PMC3867212; doi:10.1371/journal.pcbi.1003334)
Supplement: Table S4 — NKT cell-related parameters. (DOCX) [file pcbi.1003334.s022.docx]

| **Parameter** | **Value** | **Description** |
| --- | --- | --- |
| NKTCD1dAct | 0.115 | controls the CD1d-dependent activation of NKT cells |
| NKTDeact | 0.3 (from [6]) | controls the time-dependent deactivation of NKT cells |
| NKTArr | 0.3 | controls the inflow of NKT cells |
| NKTDA | 0.06 | controls the deactivation of KCs by NKT cells |
| NKTIL-4Prod | 0.2 | controls the IL-4 production of NKT cells |
| NKTIFNgProd | 3 | controls the IFN*γ* production of NKT cells |
| NKTChem | 0.1 | controls the chemokinetic effect of NKT cells on non-resident macrophages |
| NKTLife | 400 (from [7]) | half-life of NKT cells in the liver |
