# Supplementary material for: A Petri Net Model of Granulomatous Inflammation: Implications for IL-10 Mediated Control of Leishmania donovani Infection
Source: PLoS Comput Biol. 2013 Nov 21;9(11):e1003334. doi: 10.1371/journal.pcbi.1003334 (PMC3867212; doi:10.1371/journal.pcbi.1003334)
Supplement: Table S5 — NK cell-related parameters. (DOCX) [file pcbi.1003334.s023.docx]

| **Parameter** | **Value** | **Description** |
| --- | --- | --- |
| NKMacAct | 0.0005 | controls the macrophage-mediated activation of NK cells |
| IL-12NKAct | 0.0001 | controls the IL-12-mediated activation of NK cells |
| NKDeact | 0.3 (from[6]) | controls the time-dependent deactivation of NK cells |
| IL-12NKDeac | 0.1 | controls the IL-12-dependent deactivation of NK cells |
| NKArr | 0.08 | controls the inflow of NK cells |
| NKCytProd | 3 | controls the cytokine production of NK cells |
| NKEvol | 500 (from[8]) | controls the time needed for the phenotypic change of NK cells from IFN*γ*+ IL-10*−* to IFN*γ*+ IL-10+ |
| NKLife | 400 (from[7]) | half-life of NK cells in the liver |
