# Supplementary material for: A Petri Net Model of Granulomatous Inflammation: Implications for IL-10 Mediated Control of Leishmania donovani Infection
Source: PLoS Comput Biol. 2013 Nov 21;9(11):e1003334. doi: 10.1371/journal.pcbi.1003334 (PMC3867212; doi:10.1371/journal.pcbi.1003334)
Supplement: Table S6 — T cell-related parameters. (DOCX) [file pcbi.1003334.s024.docx]

| **Parameter** | **Value** | **Description** |
| --- | --- | --- |
| SpleenTCellArr | 0.05 | controls the arrival of T cells from the periphery |
| LiverTCellArr | 0.05 | controls the arrival of T cells from other granulomas in the liver |
| TCellDiff | 0.05 | controls the differentiation of T cells into subpopulations |
| TimeTStart | 96 (from [9]) | times (in hours) of the arrival of T cells from the spleen and liver |
| TimeTStop | 125 | times (in hours) when T cells stop arriving from the spleen and liver |
| TRep | 0.002 | controls the reproduction rate of T cells |
| TCellAct | 0.0005 | controls the MHC-dependent activation of T cells |
| Th1IL-10RepMod | 0.75 | controls the reduced reproduction rate of IFN*γ^+^*IL-10^+^ T cells |
| Th1IL-10Diff | 0.0001 | controls the probability of the phenotypic change in T cells from IFN*γ^+^*IL-10^-^ to IFN*γ^+^*IL-10^+^ |
| TDeact | 0.3 (from [6]) | controls the time-dependent deactivation of T cells |
| TChem | 0.1 | controls the chemokinetic effect of T cells on non-resident macrophages |
| ToZeroProd | 0.001 | controls the silencing of active cells |
| FromZeroProd | 0.001 | controls the activation of silent cells |
| Th1Evol | 120 (from [1]) | control the “transition” of IFN*γ^+^IL-*10^-^ T cells, from IFN*γ* consumption to IL-12 consumption |
| TCellKeepProd | 0.4 | controls the cytokine consumption of T cells |
| TCellCytProd | 3 | controls the cytokine production of T cells |
