# Supplementary material for: A Petri Net Model of Granulomatous Inflammation: Implications for IL-10 Mediated Control of Leishmania donovani Infection
Source: PLoS Comput Biol. 2013 Nov 21;9(11):e1003334. doi: 10.1371/journal.pcbi.1003334 (PMC3867212; doi:10.1371/journal.pcbi.1003334)
Supplement: Table S8 — P-values for LDU means equality in [13] and scaled [11] . (DOCX) [file pcbi.1003334.s026.docx]

| **Week** | **P-Value** |
| --- | --- |
| 2 | 0.4123 |
| 4 | 0.3564 |
| 8 | 0.0002 |
