# Supplementary material for: A Petri Net Model of Granulomatous Inflammation: Implications for IL-10 Mediated Control of Leishmania donovani Infection
Source: PLoS Comput Biol. 2013 Nov 21;9(11):e1003334. doi: 10.1371/journal.pcbi.1003334 (PMC3867212; doi:10.1371/journal.pcbi.1003334)
Supplement: Table S9 — P-values for LDU means equality in vivo and in silico . (DOCX) [file pcbi.1003334.s027.docx]

| **Week** | **P-value** |
| --- | --- |
| 2 | 0.15998124 |
| 3 | 0.24335254 |
| 4 | 0.59576153 |
| 8 | 0.07813823 |
| 10 | 0.005230354 |
