# Supplementary material for: A Petri Net Model of Granulomatous Inflammation: Implications for IL-10 Mediated Control of Leishmania donovani Infection
Source: PLoS Comput Biol. 2013 Nov 21;9(11):e1003334. doi: 10.1371/journal.pcbi.1003334 (PMC3867212; doi:10.1371/journal.pcbi.1003334)
Supplement: Table S10 — P-values for NKT cells number means equality in vivo and in silico . (DOCX) [file pcbi.1003334.s028.docx]

| **Day** | **P-value** |
| --- | --- |
| 1 | 0.46900689 |
| 3 | 0.16802500 |
| 7 | 0.20654284 |
| 14 | 0.04942487 |
