# Supplementary material for: A Petri Net Model of Granulomatous Inflammation: Implications for IL-10 Mediated Control of Leishmania donovani Infection
Source: PLoS Comput Biol. 2013 Nov 21;9(11):e1003334. doi: 10.1371/journal.pcbi.1003334 (PMC3867212; doi:10.1371/journal.pcbi.1003334)
Supplement: Table S11 — P-values for percentage of IFN γ+ IL-10− cells means equality in vivo and in silico . (DOCX) [file pcbi.1003334.s029.docx]

| **Day** | **P-value** |
| --- | --- |
| 21 | 0.3944316 |
| 38 | 0.9942810 |
