# Supplementary material for: A Petri Net Model of Granulomatous Inflammation: Implications for IL-10 Mediated Control of Leishmania donovani Infection
Source: PLoS Comput Biol. 2013 Nov 21;9(11):e1003334. doi: 10.1371/journal.pcbi.1003334 (PMC3867212; doi:10.1371/journal.pcbi.1003334)
Supplement: Table S15 — Percentage of helper T cells. (DOCX) [file pcbi.1003334.s033.docx]

| **Day** | **% CD4^+^IFN*γ^+^*IL10^-^** | **% CD4^+^IFN*γ^+^*IL10^+^** |
| --- | --- | --- |
| 0 | 3 *±* 0*.*75 | 0*.*12 *±* 0*.*01 |
| 21 | 12*.*14 *±* 0*.*71 | 1*.*03 *±* 0*.*23 |
| 38 | 9*.*75 *±* 1*.*87 | 0*.*38 *±* 0*.*07 |
