# Supplementary material for: A Petri Net Model of Granulomatous Inflammation: Implications for IL-10 Mediated Control of Leishmania donovani Infection
Source: PLoS Comput Biol. 2013 Nov 21;9(11):e1003334. doi: 10.1371/journal.pcbi.1003334 (PMC3867212; doi:10.1371/journal.pcbi.1003334)
Supplement: Text S1 — Petri Nets. (DOCX) [file pcbi.1003334.s034.docx]

# Text S1. Petri nets

**Figures S1 to S7** depict the Petri nets for the different entities. **Figure S1** reports only the transitions among the different entities. **Figures S2 to S7** depict the Petri nets of the different entities. In these figures, black indicates local transitions, while blue transitions span across different entities. Different edge types indicate different dynamics:

- A continuous line with a standard arrowhead indicates a standard arc. This arc *takes* tokens from the input places and *moves* tokens to the output place
- A dotted line with a standard arrowhead indicates a modifier arc. This arc does not affect the token content of a place and does not enable or disable transitions. A modifier arc is used to indicate that the number of tokens of a place is used in the *evaluation of the rate* of a transition
- A continuous line with a full circle indicates a read arc. This arc does not affect the token content of places, but *enables* the target transition only if the appropriate number of tokens is present in the input place.
- A continuous line with an empty circle indicates an inhibitor arc. This arc does not affect the token content of places, but *disables* the target transition if the appropriate number of tokens is present in the input place.

In accordance with the standard theory of stochastic Petri nets, transitions fire after a time delay randomly sampled from a negative exponential probability density function. This choice has important consequences on the efficiency of the simulation since it allows the use of the Gillespie algorithm [10].
