# Supplementary material for: A Petri Net Model of Granulomatous Inflammation: Implications for IL-10 Mediated Control of Leishmania donovani Infection
Source: PLoS Comput Biol. 2013 Nov 21;9(11):e1003334. doi: 10.1371/journal.pcbi.1003334 (PMC3867212; doi:10.1371/journal.pcbi.1003334)
Supplement: Text S2 — Dynamics of modeled entities. (DOCX) [file pcbi.1003334.s035.docx]

# Text S2. Dynamics of Modeled Entities

## Cytokines

Five cytokines have been modeled: IL-2, IL-4, IL-10, IL-12, and IFN*γ*. All the cytokines are subject to the same exponential decay, which models the diffusion and drift due to blood flow in the liver. Each cytokine is characterized by an ‘effectiveness’; the higher the effectiveness, the faster a cell will react to that cytokine. Although a true value for effectiveness cannot be determined from in vivo studies, we have attributed effectiveness to different cytokines based on certain dominance characteristics observed in the literature (e.g. IL-10 often supressing the function of IFN*γ* [32,33].

## Kupffer cells

Kupffer Cells (KCs) are characterized by their level of classical activation (KCCA), alternative activation (KCAA) and deactivation (KCDA). All the (de)activation types down-regulate each other pairwise by the removal of one token in each of the down-regulating (de)activations. The down-regulation is proportional to the number of tokens in the (de)activations involved, and to the parameter ActFight. All the (de)activations decay exponentially with the same rate. KCCA is increased by consuming IFN*γ*, the rate of increase is proportional to the parameter CytAct and to the concentration and effectiveness of IFN*γ*. KCAA is increased by consuming IL-4, the rate of increase is proportional to the parameter CytAct and to the concentration and effectiveness of IL-4. KCDA is increased by consuming IL-10, the rate of increase is proportional to the parameter CytAct and to the concentration and effectiveness of IL-10.

The effect of CD47-SIRP*α* ligation is modeled by a transition that increases KCDA with a rate proportional with the number of activated NKT cells. Finally, KCDA is increased by a transition whose rate is proportional to the number of parasites.

KCs produce IL-12 with a rate proportional to the number of KCs and to their level of classical activation. Moreover, KCs produce IL-10 with a rate proportional to the number of KCs and to their level of alternative activation and deactivation.

The initial number of KCs in a granuloma can vary randomly between 1 and 11. Additionally, a low inflow of KCs is modeled.

KCs kill the parasites with a rate proportional to KCCA, to the number of KC and to the number of parasites. Proportionality to the number of parasites is a consequence of the assumption that the killing of parasites is a parallel activity.

## Non-resident myeloid cells

Non-resident myeloid cells (monocytes, DC) have an inflow, which is proportional to the number of activated TH1 cells, activated TC1 cell and activated NKT cells (modeling the effect of chemokines), and an outflow which is inversely proportional to the number of parasites.

Non-resident myeloid cells are characterized by levels of (de)activation with the same dynamics as KCs with respect to cytokines, and exponential decay. However, parasites and NKT cells do not contribute to the deactivation of non-resident macrophages, as they do not readily internalize parasites. Neutrophils are scarce in *L. donovani* granulomas and were not modeled.

## Leishmania donovani

Only internalized *L. donovani* amastigotes were modeled (LDs). Under infective conditions, 4 LDs were assumed as the infected focus for each granuloma. The reproduction of LDs is logistic with parameter

$$LDRep \cdot LD \cdot\left( 1-\frac{LD}{KCCC\cdot KC} \right)$$

where LDRep is the reproduction rate, KC is the number of Kupffer cells and KCCC is the carrying capacity of a KC (the number of LDs that each KC can sustain). Parasites kill KCs with a rate proportional to the ratio LD/KC, and therefore to the parasite burden per KC.

## MHCI, MHCII and CD1d peptides

KCs produce MHCI-, MHCII- and CD1d- peptide complexes, with different rates proportional to different parameters and to the number of LDs. Additionally, the production of MHCII is promoted by the level of classical and alternative activation, and decreased by the level of deactivation. The peptides decay exponentially, with half-life MHCILife, MHCIILife, and CD1dLife.

## T cells

Two types of LD-specific T cells are modeled: CD8^+^ (“cytotoxic”) and CD4^+^ (“helper”). All the T cells can be either: inactive, silent, or active. Only active T cells produce cytokine and reproduce. Inactive cells become active by recognizing peptide (MHC I for CD8^+^ and MHC II for CD4^+^). Active cells consume a “keep-active” cytokine (specified below) to keep the production of cytokine active, and become silent with a probability inversely proportional to the concentration of the keep-active cytokine. Silent cells become active with a probability proportional to the concentration of the keep-active cytokine. Inactive cells have an exponentially decay which models outflow. Active and silent cells deactivate after an exponentially distributed time. T cells reproduce into inactive cells of the same population and the reproduction rate is proportional to the concentration of the keep-alive cytokine.

Biological data indicate that IFN*γ^+^*IL-10*^-^* CD4^+^ T cells require different cytokines to sustain the expression of their main transcription factor (T-bet), and thus to keep producing cytokines [1]. During the initial 4 days of infection, IFNγ is the main promoter of T-bet. However, subsequently, that role passes to IL-12. To account for this, two populations of IFNγ^+^ IL-10^−^ CD4^+^ T cells have been modeled.

While it is well-known that some CD4^+^ T cells display an IFN*γ^+^*IL-10^+^ phenotype [2], the factors that contribute to the emergence of these cells are currently an active field of research, and we assumed that IFN*γ^+^*IL-10^+^ CD4^+^ T cells evolve from IL-12-consuming IFN*γ^+^*IL-10^-^ CD4^+^ T cells. The rationale behind this assumption is that IFN*γ^+^* T cells have three phases:

1. a self-sustained phase in which they produce and consume IFN*γ*
2. an externally-sustained phase in which they produce IFN*γ* but consume IL-12 (which is produced by other leukocytes such as macrophages)
3. a self-controlled phase in which they produce both IFN*γ* and IL-10

Three populations of CD8+ T cells are modeled:

- TC0, which produce IL-2; and consume IL-2 as the keep-active cytokine
- TC1, which produce IL-2 and IFNγ; and consume IFNγ as the keep-active cytokine
- TC2, which produce IL-4 and IL-10; and consume IL-4 as the keep-active cytokine
- Inactive and active TC0 cells have a probability proportional to IFNγ and inversely proportional to IL-10 to become TC1 cells, and a probability proportional to IL-4 and inversely proportional to IL-10 to become TC2 cells.

Five populations of CD4+ are modeled:

- TH0, which produce IL-2; and consume IL-2 as the keep-active cytokine
- TH1^I^, which produce IL-2 and IFNγ; and consume IFNγ as the keep-active cytokine
- TH1^II^, which produce IL-2 and IFNγ; and consume IL-12 as the keep-active cytokine
- TH1^III^, which produce IL-2, IFNγ, and IL-10; and consume IFNγ as the keep-active cytokine
- TH2, which produce IL-4 and IL-10; and consume IL-4 as the keep-active cytokine

Inactive and active TH0 cells have a probability proportional to the level of IFNγ and inversely proportional to the level of IL-10 to become TH1^I^ cells, and a probability proportional to the level of IL-4 and inversely proportional to the level of IL-10 to become TH2 cells. Active TH1^I^ cells differentiate to active TH1^II^ cells after an exponentially distributed time. The probability of a TH1^II^ cell differentiating to an active TH1^III^ cell is proportional to the concentration of IL-12.

Since TH1^II^ cells are probably quite old, and therefore less likely to reproduce, their reproduction ability have been limited with respect to the other T cells.

T cells reach the granuloma as TC0 cells or TH0 cells, from the periphery and from the liver (modeling the migration from other granulomas). To account for the limited availability of stay-alive signals and the limited size of the granuloma microenvironment, the total number of T cells is limited to 200/granuloma. When this number is reached, no reproduction or inflow is allowed. This value reflects data from intravital imaging (6).

## Natural killer cells

Natural killer cells can be in a homeostatic (hNK) or activated state (aNK). hNK cells have a constant rate of arrival when at most 1 hNK cell is present. Additionally, hNK cells are subject to an exponential death and outflow. The probability for an hNK cell to activate is proportional to the number of LDs and to the quantity of IL-12. aNK cells deactivate, becoming hNK cells, after an exponentially distributed time, or as a consequence of low concentration of IL-12, with the probability of deactivation inversely proportional to the concentration of IL-12. aNK cells produce IFN*γ*. With time, aNK cells can evolve to aNKIL-10 cells, which produce both IFN*γ* and IL-10. aNKIL-10 cells deactivate to become hNKIL-10 cells with the same rules of aNK cells. hNKIL-10 cells are subject to the same exponential death and outflow as hNK cells.

## Natural Killer T cells

Natural Killer T cells can be in a homeostatic (hNKT) or activated state (aNKT). When at most 3 hNKT cells are present, hNKT cells have a constant rate of arrival; otherwise no inflow is present. Additionally hNKT cells are subject to an exponential death and outflow. For simplicity, hNKT cells are activated (becoming aNKT cells) by consuming a CD1d peptide. aNKT cells are deactivated (returning to being hNKT cells), after an exponentially distributed time. aNKT cells produce IFN*γ* and low levels of IL-4.

## Parameters of the model

**Tables S1 to S6** describe the name, value and role of the different parameters used.
