# Supplementary material for: A Petri Net Model of Granulomatous Inflammation: Implications for IL-10 Mediated Control of Leishmania donovani Infection
Source: PLoS Comput Biol. 2013 Nov 21;9(11):e1003334. doi: 10.1371/journal.pcbi.1003334 (PMC3867212; doi:10.1371/journal.pcbi.1003334)
Supplement: Text S3 — Statistical analysis. (DOCX) [file pcbi.1003334.s036.docx]

# Statistical analysis

The means of the populations were compared using the Student’s t test for equality with the correction of Welch’s for the degree of freedom. Since the available literature reports largely different parasite burdens (LDU) even under wild type conditions, the data were scaled by minimizing the distance from [11]. The simplification underlying this approach is that for every couple of LDU distribution under wild type condition *X* (*t*) and *Y* (*t*) then

$$Y\left( t \right)=\alpha X(t)$$

and therefore

$$Var(Y\left( t \right))=\alpha^{2}Var(Y\left( t \right))$$

This rather strong simplification allows the comparison of gene-knockout models obtained from articles with different parasite burdens under wild type conditions. The value from [12] and [11] where multiplied by 0.8268 and 0.4626 respectively. **Tables S7 and S8** indicates that this procedure leads to reasonably comparable means. **Tables S9-S14** indicate that the comparison of the mean of *in silico* and *in vivo* data generally results in large p-values thus supporting the good compatibility of the model with biological values.
