# Supplementary material for: A Petri Net Model of Granulomatous Inflammation: Implications for IL-10 Mediated Control of Leishmania donovani Infection
Source: PLoS Comput Biol. 2013 Nov 21;9(11):e1003334. doi: 10.1371/journal.pcbi.1003334 (PMC3867212; doi:10.1371/journal.pcbi.1003334)
Supplement: Text S4 — Sensitivity analysis. (DOCX) [file pcbi.1003334.s037.docx]

# Text S4. Sensitivity Analysis

In generating this model, the various entities were extensively parameterized. Some parameters were extracted directly from the biological literature or from unpublished data and therefore have a straightforward biological significance. However, other parameters result from modeling decisions and simplifications and were determined by fitting the data.

For example, the probability of activation of a T cell is not biologically determinable, as the range of peptides available for presentation to T cells that are derived from *Leishmania* parasites is yet to be completely characterized, and the responsiveness of T cells to such peptide largely varies as a function of similarly ill-defined variation in T cell receptor affinity.

The variability in parameter values that results from biological diversity is common to most biological processes (e.g., see [4] for a discussion on the variability of the half-life of MHC II peptides). To account for this variability and for the subjectivity of fitted parameters, we performed sensitivity analysis to assess the robustness of the model. Following established methodology [14], we sampled the parameter space using Latin hypercube sampling and studied the impact of each parameter on the parasite burden using Partial Rank Correlation Coefficients (PRCC). A detailed description of the parameters and their values, along with the range of variation and the reference literature, is available below.

Wherever possible, the range of variation studied was related to known biological variability. Given the stochastic nature of the model, a dummy parameter, with no effect on the parasite burden, was included in the analysis. The PRCC of this dummy parameter provided a way of estimating the effect of the intrinsic model stochasticity: parameters with PRCC comparable with that of the dummy parameter, or lower, have a limited impact which is likely lower that the intrinsic model stochasticity. A total of 1800 model runs were used to perform sensitivity analysis.

The PRCC of the parameters for the parasite burden around three important stages of EVL: 1) the beginning of the adaptive immune response (100h), the peak parasite burden (500h) and the final stages of immune response (1000h) is shown in **Figure S8.** A positive (respectively negative) PRCC value for a particular parameter indicates that increasing (resp. decreasing) the value of that parameter leads to an increased (resp. decreased) parasite burden at the times indicated above.

**Figures S9 to S16** display the PRCC of some of the most important parameters. Parameters with PRCC significantly larger in magnitude that the dummy parameters should be considered important for the model. See the caption of the figures for comments on the single parameters.
