# Supplementary material for: A Petri Net Model of Granulomatous Inflammation: Implications for IL-10 Mediated Control of Leishmania donovani Infection
Source: PLoS Comput Biol. 2013 Nov 21;9(11):e1003334. doi: 10.1371/journal.pcbi.1003334 (PMC3867212; doi:10.1371/journal.pcbi.1003334)
Supplement: Text S5 — Assumptions and simplifications of the model. (DOCX) [file pcbi.1003334.s038.docx]

# Text S5 Assumptions and simplifications of the model

This section documents the most important assumptions and simplification used by the model. Specific references are presented where available.

**Assumption S1.** *All the cytokines of the same type are identical.*

While this may seem obvious, some biological evidence suggests otherwise. For instance, [15] reports that in the early stages of granuloma formation, macrophages react to IFN*γ* produced by NKT cells, but not to IFN*γ* produced by other liver cells.

**Assumption S2.** *The rate of reproduction of Leishmania donovani amastigotes is the same in both (de)activated and normal macrophages.*

**Assumption S3.** *All Leishmania donovani amastigotes behave in the same way.*

This implies that individual differences among amastigotes can be ignored.

**Assumption S4.** *Leishmania donovani amastigotes interact with Kupffer cells in the same way at different stages of reproduction.*

**Assumption S5.** *Leishmania donovani amastigotes promote the deactivation of the Kupffer cell that ingested them.*

While no clear evidence of this assumption exists, experiments indicate that macrophages infected with *Leishmania donovani* produce IL-10 [16] and display reduced MHC expression [17], both activities are associated with deactivated macrophages.

**Assumption S6.** *The alteration of Kupffer cell activities by Leishmania donovani is proportional to the number of parasites ingested by the Kupffer cell.*

**Assumption S7.** *Proliferation of Kupffer cells is mostly negligible during leishmaniasis.*

**Assumption S8.** *The cytokine production of macrophages is as follows:*

*• Classically activated macrophages mainly produce TNF and IL-12.*

*• Alternatively activated macrophages mainly produce IL-10.*

*• Deactivated macrophages mainly produce IL-10.*

While (de)activated macrophages have been reported to produce a number of different types of cytokines [18], the above are the cytokines produced in largest quantities.

**Assumption S9.** *Classical activation, alternative activation, and deactivation coexist, but down-regulate each other.*

Some studies suggest that in macrophages the different types of activation can coexist (see for example [19]). The down-regulation is a consequence of the possible internal reactions and of the antagonizing effects of the cytokines produced.

**Assumption S10.** *The (de)activation of macrophages is as follows:*

- *Classical activation of macrophages is triggered by TNF and IFN*
- *Alternative activation of macrophages is triggered by IL-4 and IL-13.*
- *Deactivation of macrophages is triggered by IL-10.*

It is generally recognized that (de)activation of macrophages is triggered by the above cytokines [18].

**Assumption S11.** *When infected, macrophages produce TNF.*

Infected macrophages produce TNF. The production of TNF subsequently increases the activity of macrophages in an autocrine way.

**Assumption S12.** *Multinucleated giant cells do not have specific effect on the behavior of liver granulomas.*

We assume that a multinucleated giant cells with *n* nuclei is functionally equivalent to a group of *n* macrophages. This rather strong assumption is due to the limited knowledge on the effect of macrophage fusion in the context of *Leishmania*-induced granulomas. Moreover, even considering granuloma formed in response to tuberculosis, there is no clear evidence indicating whether the role of multinucleated cells is positive or negative. Finally, liver granulomas in EVL are quite small, and the number of multinucleated giant cells is generally low.

**Assumption S13.** *Kupffer cells need to be classically activated to kill Leishmania donovani amastigotes.*

Experiments indicate that classical activation is fundamental to kill *Leishmania* amastigotes. Moreover, the efficiency in killing the parasites depends on the level of classical activation, and thus on the level of external stimuli promoting classical activation.

**Assumption S14.** *NKT cells deactivate infected Kupffer cells via a juxtacrine mechanism.*

**Assumption S15.** *The only subsets of T cells that impact on the formation and maintenance of a granuloma are TH0, TH1, TH2, TC 0, TC1, and TC*2 *cells.*

While a number of other subsets of T cells exist, such as TH17 cells, the cell populations mentioned above appear to be the most numerous in EVL granulomas.

**Assumption S16.** *TH0, TH1, TH2, TC0, TC1, and TC2* *cell populations exist and are stable.*

It is common to classify the populations of CD4^+^ and CD8^+^ T cells by the cytokines they produce. However, some authors are beginning to question this classification, or at least to point out that it is not *strict*. This is due to the large variability that seems to exist within the same population, and to the plasticity of T cells (see for example [20]).

**Assumption S17.** *TH0* *cells differentiate to TH1* *cells when exposed to IFNγ (and later IL-12).*

The process of differentiation of TH0 cells into TH1 cells is believed to be triggered by IFN*γ* and IL-12. However, recent articles describe how the activity of T-bet is promoted by IFN*γ* and IL-12 independently [1].

**Assumption S18.** *TC0* *cells differentiate to TC1* *cells when exposed to IFNγ.*

IFN*γ* is believed to be the main cytokine responsible for the differentiation of TC0 cells to TC1 cells.

**Assumption S19.** *TH0* *and TC0* *cells differentiate to TH2* *and TC2* *cells when exposed to IL-4.*

IL-4 is believed to be the main cytokine responsible for the differentiation of TH0 and TC0 cells to TH2 and TC2 cells, respectively.

**Assumption S20.** *Newly differentiated TH1* *and TC1* *cells produce mainly IFNγ and IL-2.*

TH1 and TC1 cells produce different types of cytokines. However, newly differentiated TH1 and TC1 cells produce mainly IFN*γ* (responsible for the initial differentiation driven expansion of the cell population) and IL-2 (responsible for the expansion of all T cells subtypes).

**Assumption S21.** *TH1 cells initiate the production of IL-10 after the second wave of T-bet expression caused by IL-12.*

As described in [2], TH1 cells display *self-control* by producing IL-10. This phenomenon is clearly visible in an established liver granuloma where 2 – 5 % of T cells produce IFN*γ* and IL-10 (unpublished data). Different factors have been proposed to be responsible for this phenotypic change, and no evidence clearly indicates when this change happens. However, it is reasonable to divide the response of TH1 cells into three phases. In the first phase, their activity is auto-promoted by IFN*γ*. In the second phase, their activity is promoted by IL-12. Since IL-12 is not produced by TH1 cells, they need to be sustained *externally*. In the third phase, besides requiring IL-12, TH1 cells initiate the production of IL-10 to self-control their activity.

**Assumption S22.** *TH*2 *and TC*2 *cells mainly produce IL-4 and IL-10.*

TH2 cells produce different types of cytokines. However, they mainly produce IL-4 and IL-10.

**Assumption S23.** *IFNγ promotes the proliferation/differentiation of TH2 and TC2 cells.*

IFN*γ* is commonly considered to be a promoter of the proliferation / differentiation of TH1 and TC1 cells.

**Assumption S24.** *IL-4 promotes the proliferation / differentiation of TH2* *and TC2* *cells.*

IL-4 is commonly considered to be a promoter of the proliferation / differentiation of TH2 and TC2 cells.

**Assumption S25.** *NKT cells are important for the formation of granulomas (specifically before the arrival of effector T cells).*

The role of NKT cells in the early stages of granuloma formation has been extensively confirmed (e.g., [21] and [11]).

**Assumption S26.** *NKT cells mainly produce high quantities of IFNγ and low quantities of IL-4 in response to Leishmania donovani infection.*

As described for example in [22], NKT cells can produce a broad range of cytokines. However, IL-4 and IFN*γ* appear to be the cytokines produced in larger quantities. Experiments indicate that IL-4 production by NKT cells is low during the response to liver infection by *Leishmania donovani*.

**Assumption S27.** *NKT cells detect an infected macrophage and become activated.*

NKT cells can be activated by a number of mechanisms [22]. However, peptides displayed by CD1d seem to have a fundamental role in leishmaniasis [21].

**Assumption S28.** *NKT cells remain active for some time and then deactivate.*

It is currently unclear if the deactivation of NKT cells is *time-controlled*. But their lineage connection with T cells makes this assumption plausible.

**Assumption S29.** *At homeostasis, the liver population of NKT cells is stable, but they have a high turnover rate.*

The exact mechanism behind the stability of the number of homeostatic NKT cells in the liver is not well- understood. “The number of NK1.1^+^ T cells found in the liver of adult mice did not steadily increase. These results suggest that liver NK1.1^+^ T cells have a high death rate, migrate from the liver, or undergo a phenotypic change. BrdU data suggests either that NK1.1^+^ T cells begin proliferating after emigration from the thymus to the liver, or that the liver is populated by a special set of thymic NK1.1^+^ T cells, which are proliferating or were recently generated from proliferating precursors [23]”.

**Assumption S30.** *The population of homeostatic NKT cells is stable, even when an infection is present.*

It is not possible to test this assumption with the current technology. During leishmaniasis, the total number of hepatic NKT cells increases [11], so we have assumed that these additional NKT cells transition to become activated NKT cells.

**Assumption S31.** *Peripheral blood reproduction of NKT cells is negligible.*

No clear evidence exists for peripheral blood reproduction of NKT cells. Therefore, we will disregard the phenomenon. Increased NKT cell number in the liver during infection may arise from influx from the periphery.

**Assumption S32.** *NK cells produce IFNγ in response to Leishmania donovani infection.*

The exact mechanism used by NK cells to recognize infected cells is not completely understood. However, they have been shown to be a source of IFN*γ* during leishmaniasis. Possible sources of activation are IL-12 [21] or direct recognition of infected macrophages.

**Assumption S33.** *NK cells produce high levels of IL-10 in the later stages of granuloma development.*

[8] reports that, while NK cells produce mainly IFN*γ* in the early stages of granuloma, later on they initiate the production of IL-10. A sustained production of IL-10 is reported 21 days after infection.

**Assumption S34.** *Peripheral blood reproduction of NK cells is negligible.*

No clear evidence exists for peripheral blood reproduction of NK cells. Therefore, we will disregard the phenomenon. Increased NK cell number in the liver may result from influx from the periphery.

**Assumption S35.** *Death of activated NK cells can be ignored.*

No clear evidence exists on the expected life of an activated NK. However, the data of [8] reports that NK cells synthesize IL-10 mRNA for about 21 days before initiating the production of IL-10. A possible explanation is that activated NK cells synthesize IL-10 mRNA long before initiating the production of IL-10. This suggests that activated NK cells survive for a long time (at least 21 days).

**Assumption S36.** *NK cells deactivate after a fixed probabilistic time.*

The mechanism behind the (de)activation of NK cells are not completely understood. Given the limited role that NK cells seem to play in leishmaniasis we opted for a simple model of deactivation (similar to T and NKT cells).

**Assumption S37.** *Leishmania donovani amastigotes take about 1 day to reproduce.*

*In vitro* experiments indicate that the reproduction cycle of *Leishmania donovani* amastigotes is about 24 hours.

**Assumption S38.** *The half-life of MHCI - peptide complexes is 3 hours.*

The half-life of MHC I peptides is generally reported to be either 3 or 6 hours [3]. We used the value 3, but characterized the effect of increasing it to 6 by sensitivity analysis.

**Assumption S39.** *The half-life of MHCII - peptide complexes is 60 hours.*

The literature indicates a large variability in the half-life of MHC II - peptide complexes (see for example [24]), and it difficult to characterize the full spectrum of peptides displayed by antigen-presenting cells in response to *Leishmania donovani* infection (see [25] for some data). However, MHC II - peptide complexes are generally believed to be quite stable and their half-life is believed to be in the order of days. The chosen value (60 hours) was also used by [4] (albeit in a different context) and should be a reasonable estimate.

**Assumption S40.** *The half-life of CD1d - peptides complexes is 20 hours.*

CD1d/*α*-galactosylceramide complexes are probably the most well known CD1d-peptide complexes. However, the reported half-life varies between few minutes and few days [5]. We used a baseline value of 20 hours to account for the fact that the complexes are believed to be quite stable.

**Assumption S41.** *Kupffer cells can sustain about 100 amastigotes.*

Direct observations of granulomas indicate the presence of macrophages with more than 100 amastigotes *in vivo*. This number is likely affected by many external conditions, as during *in vitro* experiments the number of amastigotes that a macrophage can sustain before being killed is about 50. 100 should be a reasonable mean value.

**Assumption S42.** *Activated Kupffer cells can kill amastigotes in about 1 day.*

*In vitro* experiments indicate that strongly activated macrophages are able to kill 10 parasites in 24-48 hours. However, this activity is likely to be strongly parallel.

**Assumption S43.** *T cells get to the liver 4 days after infection.*

This value has been confirmed experimentally and is compatible with the T cell dynamics described in [9]. During these 4 days various events happen:

1. Infected dendritic cells migrate to the lymph nodes, or spleen, and mature (about 16 hours)

2. Naïve T cells with a matching TCR contact with the mature dendritic cell (about 1 day)

3. The naïve T cells mature and replicate (probably at least 53 hours, as this is the time needed by a naïve T Cell to reach the fifth generation and therefore its full cytokine production profile [26])

4. The T cells migrate to the liver (few hours)

**Assumption S44.** *Spleen-derived T cells stop arriving in the liver 5 days after infection.*

The half-life of mature dendritic cells is not fully characterized yet. However, a too long half-live would lead to an excessive immune response. A 1-day influx of cells from the spleen seemed a reasonable value. Note that, given the peripheral blood reproduction of T cells, and the influx of mature T cells from other granulomas, this value appeared not to be worth an extensive investigation.

**Assumption S45.** *Activated TH1* *cells require IFNγ to sustain the production of cytokines in the first 5 days, and IL-12 subsequently.*

[1] indicates that IFN*γ* and IL-12 sustain two distinct waves of T-bet expression. The two waves peak one and five days after activation respectively.

**Assumption S46.** *When the TCR of a T cell recognizes appropriate MHC-peptide complexes on a macrophage, the cells interact for approx. 30 minutes.*

This result is from [6] and is relative to CD8^+^ cells.

**Assumption S47.** *The percentage of cytokine producing helper T cells follows* ***Table S9****.*

These data are generated, and are currently unpublished. **Table S9** indicates the mean value and the standard deviation. The value at day 0 and the percentage of NKT cells from [21] were used to calculate a fixed number of *Leishmania donovani* non-specific activated helper T cells (assuming the same number of CD4^+^ and CD8^+^ T cells).

**Assumption S48.** *The half-life of mature NK cells in the liver is about 400 hours (about 17 days).*

[27] reports that “the half-life of mature NK cells in the periphery appears to be about seven to ten days based on the survival of adoptively transferred NK cells”. However, the half-life of splenic NK cells is reported to be 17 days [7] and this value was used.

**Assumption S49.** *NK cells initiate the production of IL-10 after 21 days of activation.*

The experiments reported by [8] indicate that NK cells are sources of IL-10 21 days after infection.

**Assumption S50.** *The half-life of mature NKT cells in the liver is about 400 hours (about 17 days).*

Since we were not able to find any data on the half-life of liver NKT cells, given the connection between NK and NKT cells, we used the same value of NK cells (see Assumption 48)

**Assumption S51.** *The number of granulomas in the liver is about 5x10^5^.*

This estimate is obtained from intravital imaging and volume reconstruction [28].

**Simplification S1.** *Fully (de)activated macrophages, activated NK cells, NKT cells, and T cells produce the same amount of cytokines.*

It is quite hard to determine the cytokine production of the various types of cells in vivo. Therefore, we set the value to be the same. Note that the level of production of the modeled cytokines produced by T cells is quite similar.

**Simplification S2.** *All the cells that produce cytokines reach a stable level of production.*

The quantity of cytokines produced by a leukocyte is not constant, and may depend on many factors, such as co-stimulatory signals. Moreover, cytokine production by T cells is likely to be affected by the affinity of their TCR with the specific peptide displayed by MHC molecules. For instance, TH1 cells reach a stable level of cytokines production after 5 cycles of reproduction [26] and to maintain that level they need a favorable environment [1].

**Simplification S3.** *The level of cytokine production of the cells is fixed.*

Many factors influence the cytokine production of T cells. However, given our modeling formalism, we used a fixed value.

**Simplification S4.** *Only one type of chemokine exists and its role is purely chemoattractive.*

While different types of chemokines exist, their effects are often similar. We assume that only one type of chemokine exists, and that chemokines affect only the arrival of non-resident macrophages. Additionally, the role of CCL3 in promoting TH1 cells - based immunity will be ignored. This is justifiable by the fact that, in our model, NKT cells, which are the only sources of CCL3, produce IFN*γ*, which is by itself a strong promoter of the differentiation of TH1 cells. We will not use any specific name for chemokines, and refer generically to “chemokines”. Moreover, we will model only the effect of chemokines on non-resident mononuclear phagocytes.

**Simplification S5.** *The chemoattractive effect of chemokines is proportional to the number of chemokine-producing cells.*

Chemokines have two fundamental roles: they facilitate the passage of leukocytes through the wall of the blood vessels and stimulate the leukocytes to move towards a place with a higher density of chemokines. We will simply assume that the level of chemokines influences the arrival rate of affected leukocytes.

**Simplification S6.** *The leishmanicidal effects of TNF are adequately modeled through IFNγ.*

TNF plays a central role in granuloma formation in EVL [29] and complete loss of TNF leads to delayed granuloma initiation, poor parasite control and ultimately the generation of a lethal inflammatory response. The precise mechanisms by which TNF affects these features of the response are unknown, and are not modeled here. Our model is concerned more with the regulation of leishmanicidal activity within fully formed granulomas, and in this context TNF works largely in cooperation with IFN*γ*.

**Simplification S8.** *Cytokine tokens represent more than one molecule.*

Determining the number of cytokine molecules produced by cells is technically very difficult. Moreover, cytokine production varies over time in accordance with the stimuli received by the cell. Cytokine tokens, which are used by our model, should be considered as a group of molecules.

**Simplification S9.** *A macrophage can undergo classical activation, alternative activation, or deactivation.*

The current biological opinion is that a macrophage is able to undergo a number of different types of activation. However, liver granulomas are not so disruptive to require a healing macrophage to repair the extracellular matrix. Moreover, classical activation, alternative activation, and deactivation are by far the most studied activation types.

**Simplification S10.** *Classically activated macrophages only produce IL-12.*

While classically activated macrophages have been reported to produce a number of different types of cytokines, IL-12 is the cytokine of most significance here.

**Simplification S11.** *Alternatively activated and deactivated macrophages only produce IL-10.*

While alternatively activated and deactivated macrophages have been reported to produce a number of different types of cytokines, IL-10 is the cytokine of most significance here.

**Simplification S12.** *Alternative activation of macrophages is triggered by IL-4 alone.*

It is generally recognized that alternative activation is triggered by IL-4 and IL-13. However, in our model, the only recognized sources of IL-13, iNKT and TH2 cells, also produce IL-4. Therefore, we assign to IL-4 the role of both IL-4 and IL-13.

**Simplification S13.** *The cognate interaction of NKT cells with infected Kupffer cells promotes deactivation of the latter.*

Since we imposed that classical activation is required for killing of *Leishmania* amastigotes (Assumption S13), we are not interested in the normal killing activity of macrophages. Therefore, increasing the deactivation of a macrophage implies a down-regulation of its killing activity.

**Simplification S14.** *The cytokine production of activated T cells is as follows:*

*• TH1* *cells produces IL-2 and IFNγ or IL-2, IFNγ and IL-10 only.*

*• TC1* *cells produces IL-2 and IFNγ only.*

*• TH2* *and TC2* *cells produce IL-4 and IL-10 only.*

**Simplification S15.** *T cells deactivate after a fixed probabilistic time.*

T cells activation is a complex and a not yet fully understood process [30]. It seems, however, possible that a T cell continues to be in active state for some time. Modeling this mechanism would have required a much more complex model of T cells (and additional parameters).

**Simplification S16.** *Apoptosis of T cells can be ignored.*

Many mechanisms regulate the activities of activated T cells, and apoptosis is one of the most important. While the network of signals that leads to apoptosis is not yet fully characterized, we know that many competing signals (see for example [31]) regulate it. Most of these signals require many cell phenotypes that are not described by our models. Therefore, activated T cells will simply not die in our model.

**Simplification S17.** *Cytokines have no effect on the deactivation of T cells.*

Many cytokines regulate the behavior of T cells, while some of them promote the emergence of a phenotype, others antagonize it. For example, IFN*γ* promotes the differentiation of TH0 cells to TH1 cells while blocking differentiation to TH2 cells. We will consider the effect of cytokines only on proliferation / differentiation, but ignore the effect on deactivation.

**Simplification S18.** *T Cells consume their flag cytokine to keep their phenotype active.*

Each T cell subpopulation produces a *flag cytokine*, that is, a cytokine that is characteristic of that subpopulation (IFN*γ* for TH1, IL-4 for TH2, and IL-2 for TH0 and TC0). The flag cytokine has a positive feedback effect on the phenotype by which it is produced and a negative feedback effect on the others. For example, IFN*γ* promotes the expansion of TH1 cells and down-regulates the expansion of TH2 cells. Moreover, from the data of [1] we know that TH1 cells need constant exposure to IFN*γ* and IL-12 to preserve their ability to produce IFN*γ*. Therefore, the producing cell itself consumes a proportion of the flag cytokine produced.

**Simplification S19.** *The cytokine production of a TH1* *cell requires IFNγ up to 120 hours after activation, and IL-12 subsequently.*

As described by [1], IFN*γ* and IL-12 stimulate the expression of T-bet independently. Given the modeling technique used, we simplified this behavior using two distinct populations. Note that, in our model, TH0 cells differentiate only to IFN*γ*-consuming TH1 cells, and only at this point IL-12 is considered as a factor for the promotion of the population of TH1 cells. This is due to the fundamental role of IFN*γ* in our model, which leads to a limited interest in a model without IFN*γ*.

**Simplification S20.** *The inflow of T cells in the granuloma is constituted only by TH0* *cells.*

Different populations of T cells enter the granuloma microenvironment. Notably, different types of TH1 cells migrate from other granulomas, and contribute to a stronger inflammatory response. Given the local nature of our model, this aspect was not modeled.

**Simplification S21.** *There is a constant influx of homeostatic NKT cells, but no reproduction.*

As described by Assumption 29, the mechanism employed by the immune system to keep the population of liver homeostatic NKT cells stable is not well characterized. Since the model we used is local, we are not able to test hypotheses on the behavior of NKT cells in the whole liver. Therefore, we are not able to test whether NKT cells reproduce in the liver or not. We will just assume that there is a constant influx of NKT cells (as documented by [23]), ignoring the source of this influx.

**Simplification S22.** *There is a constant influx of homeostatic NK cells, but no reproduction.*

Considerations similar to those described by Simplification S21, led to this simplification.
