# Supplementary material for: A Petri Net Model of Granulomatous Inflammation: Implications for IL-10 Mediated Control of Leishmania donovani Infection
Source: PLoS Comput Biol. 2013 Nov 21;9(11):e1003334. doi: 10.1371/journal.pcbi.1003334 (PMC3867212; doi:10.1371/journal.pcbi.1003334)
Supplement: Text S6 — Supplementary references. (DOCX) [file pcbi.1003334.s039.docx]

# Text S6. Supplementary references

1. Schulz EG, Mariani L, Radbruch A, Höfer T (2009) Sequential Polarization and Imprinting of Type 1 T Helper Lymphocytes by Interferon-γ and Interleukin-12. Immunity 30: 673–683. doi:10.1016/j.immuni.2009.03.013.

2. Trinchieri G (2007) Interleukin-10 production by effector T cells: Th1 cells show self control. The Journal of Experimental Medicine 204: 239–243. doi:10.1084/jem.20070104.

3. Henrickson SE, Mempel TR, Mazo IB, Liu B, Artyomov MN, et al. (2008) T cell sensing of antigen dose governs interactive behavior with dendritic cells and sets a threshold for T cell activation. Nat Immunol 9: 282–291. doi:10.1038/ni1559.

4. Linderman JJ, Riggs T, Pande M, Miller M, Marino S, et al. (2010) Characterizing the Dynamics of CD4+ T Cell Priming within a Lymph Node. The Journal of Immunology 184: 2873–2885. doi:10.4049/jimmunol.0903117.

5. Benlagha K, Weiss A, Beavis A, Teyton L, Bendelac A (2000) In Vivo Identification of Glycolipid Antigen–Specific T Cells Using Fluorescent Cd1d Tetramers. The Journal of Experimental Medicine 191: 1895–1904. doi:10.1084/jem.191.11.1895.

6. Beattie L, Peltan A, Maroof A, Kirby A, Brown N, et al. (2010) Dynamic Imaging of Experimental Leishmania donovani-Induced Hepatic Granulomas Detects Kupffer Cell-Restricted Antigen Presentation to Antigen-Specific CD8+ T Cells. PLoS Pathog 6: e1000805. doi:10.1371/journal.ppat.1000805.

7. Jamieson AM, Isnard P, Dorfman JR, Coles MC, Raulet DH (2004) Turnover and Proliferation of NK Cells in Steady State and Lymphopenic Conditions. The Journal of Immunology 172: 864–870.

8. Maroof A, Beattie L, Zubairi S, Svensson M, Stager S, et al. (2008) Posttranscriptional Regulation of Il10 Gene Expression Allows Natural Killer Cells to Express Immunoregulatory Function. Immunity 29: 295–305.

9. Murphy K, Travers P, Walport M (2007) Janeway’s Immunobiology. 7th ed. Garland Science.

10. Koch I, Reisig W, Schreiber F (2010) Modeling in Systems Biology: The Petri Net Approach. 1st ed. New York, NY, USA: Springer-Verlag New York, Inc.

11. Stanley AC, Zhou Y, Amante FH, Randall LM, Haque A, et al. (2008) Activation of Invariant NKT Cells Exacerbates Experimental Visceral Leishmaniasis. PLoS Pathog 4: e1000028. doi:10.1371/journal.ppat.1000028.

12. Murphy ML, Wille U, Villegas EN, Hunter CA, Farrell JP (2001) IL-10 mediates susceptibility to Leishmania donovani infection. European Journal of Immunology 31: 2848–2856. doi:10.1002/1521-4141(2001010)31:10<2848::AID-IMMU2848>3.0.CO;2-T.

13. Murray HW, Tsai CW, Liu J, Ma X (2006) Responses to Leishmania donovani in Mice Deficient in Interleukin-12 (IL-12), IL-12/IL-23, or IL-18. Infection and Immunity 74: 4370–4374. doi:10.1128/IAI.00422-06.

14. Saltelli A, Chan K, Scott EM (2000) Sensitivity Analysis. Wiley. 504 p.

15. Svensson M, Zubairi S, Maroof A, Kazi F, Taniguchi M, et al. (2005) Invariant NKT Cells Are Essential for the Regulation of Hepatic CXCL10 Gene Expression during Leishmania donovani Infection. Infection and Immunity 73: 7541–7547. doi:10.1128/IAI.73.11.7541-7547.2005.

16. Bhattacharyya S, Ghosh S, Jhonson PL, Bhattacharya SK, Majumdar S (2001) Immunomodulatory Role of Interleukin-10 in Visceral Leishmaniasis: Defective Activation of Protein Kinase C-Mediated Signal Transduction Events. Infection and Immunity 69: 1499–1507. doi:10.1128/IAI.69.3.1499-1507.2001.

17. Reiner NE, Ng W, McMaster WR (1987) Parasite-accessory cell interactions in murine leishmaniasis. II. Leishmania donovani suppresses macrophage expression of class I and class II major histocompatibility complex gene products. The Journal of Immunology 138: 1926–1932.

18. Mantovani A, Sica A, Sozzani S, Allavena P, Vecchi A, et al. (2004) The chemokine system in diverse forms of macrophage activation and polarization. Trends in immunology 25: 677–686.

19. Mosser DM, Edwards JP (2008) Exploring the full spectrum of macrophage activation. Nat Rev Immunol 8: 958–969. doi:10.1038/nri2448.

20. Bluestone JA, Mackay CR, O’Shea JJ, Stockinger B (2009) The functional plasticity of T cell subsets. Nat Rev Immunol 9: 811–816. doi:10.1038/nri2654.

21. Amprey JL, Im JS, Turco SJ, Murray HW, Illarionov PA, et al. (2004) A Subset of Liver NK T Cells Is Activated during Leishmania donovani Infection by CD1d-bound Lipophosphoglycan. The Journal of Experimental Medicine 200: 895–904. doi:10.1084/jem.20040704.

22. Matsuda JL, Mallevaey T, Scott-Browne J, Gapin L (2008) CD1d-restricted iNKT cells, the “Swiss-Army knife” of the immune system. Current Opinion in Immunology 20: 358–368. doi:10.1016/j.coi.2008.03.018.

23. Coles MC, Raulet DH (2000) NK1.1+ T Cells in the Liver Arise in the Thymus and Are Selected by Interactions with Class I Molecules on CD4+CD8+ Cells. The Journal of Immunology 164: 2412–2418.

24. Lazarski CA, Chaves FA, Jenks SA, Wu S, Richards KA, et al. (2005) The Kinetic Stability of MHC Class II:Peptide Complexes Is a Key Parameter that Dictates Immunodominance. Immunity 23: 29–40. doi:10.1016/j.immuni.2005.05.009.

25. Afrin F, Rajesh R, Anam K, Gopinath M, Pal S, et al. (2002) Characterization of Leishmania donovani Antigens Encapsulated in Liposomes That Induce Protective Immunity in BALB/c Mice. Infection and Immunity 70: 6697–6706. doi:10.1128/IAI.70.12.6697-6706.2002.

26. Gudmundsdottir H, Wells AD, Turka LA (1999) Dynamics and Requirements of T Cell Clonal Expansion In Vivo at the Single-Cell Level: Effector Function Is Linked to Proliferative Capacity. The Journal of Immunology 162: 5212–5223.

27. Yokoyama WM, Kim S, French AR (2004) The Dynamic Life of Natural Killer Cells. Annu Rev Immunol 22: 405–429. doi:10.1146/annurev.immunol.22.012703.104711.

28. Beattie L (2010) Personal communication.

29. Murray HW, Jungbluth A, Ritter E, Montelibano C, Marino MW (2000) Visceral Leishmaniasis in Mice Devoid of Tumor Necrosis Factor and Response to Treatment. Infection and Immunity 68: 6289–6293. doi:10.1128/IAI.68.11.6289-6293.2000.

30. Smith-Garvin JE, Koretzky GA, Jordan MS (2009) T Cell Activation. Annu Rev Immunol 27: 591–619. doi:10.1146/annurev.immunol.021908.132706.

31. Holtzman MJ, Green JM, Jayaraman S, Arch RH (2000) Regulation of T cell apoptosis. Apoptosis 5: 459–471. doi:10.1023/A:1009657321461.

32. Bogdan, C, and Nathan, C. (1993) Modulation of macrophage function by transforming growth factor beta, interleukin-4, and interleukin-10. Ann N Y Acad Sci. 685:713-39.

33. [Fiorentino DF](http://www.ncbi.nlm.nih.gov/pubmed?term=Fiorentino%20DF%5BAuthor%5D&cauthor=true&cauthor_uid=1940369), [Zlotnik A](http://www.ncbi.nlm.nih.gov/pubmed?term=Zlotnik%20A%5BAuthor%5D&cauthor=true&cauthor_uid=1940369), [Mosmann TR](http://www.ncbi.nlm.nih.gov/pubmed?term=Mosmann%20TR%5BAuthor%5D&cauthor=true&cauthor_uid=1940369), [Howard M](http://www.ncbi.nlm.nih.gov/pubmed?term=Howard%20M%5BAuthor%5D&cauthor=true&cauthor_uid=1940369), [O'Garra A](http://www.ncbi.nlm.nih.gov/pubmed?term=O'Garra%20A%5BAuthor%5D&cauthor=true&cauthor_uid=1940369). (1991) IL-10 inhibits cytokine production by activated macrophages. J Immunol. 147(11):3815-22.
